# Repurposing Artesunate to Combat Progression and Metastasis via Targeting Circulating Tumor Cells

**DOI:** 10.32604/or.2026.075600

**Published:** 2026-05-21

**Authors:** Evangelia Pantazaka, Dimitrios Papakonstantinou, Argyro Roumeliotou, Dafni Graikioti, Sotirios Tsakas, Nefeli Zacharopoulou, Stuart S. Martin, Athanasios Kotsakis, Constantinos M. Athanassopoulos, Catherine Alix-Panabières, Galatea Kallergi

**Affiliations:** 1 Laboratory of Biochemistry/Metastatic Signaling, Section of Genetics, Cell Biology and Development, Department of Biology, University of Patras, University Campus, Patras, Greece; 2 Synthetic Organic Chemistry Laboratory, Department of Chemistry, University of Patras, University Campus, Patras, Greece; 3 Medical Science Liaison MSL Oncology & Rare Diseases, Medical Department, Ipsen Greece & Cyprus, IPSEN SP LLC, 63 Agiou Dimitriou Str., Alimos–Athens, Greece; 4 Department of Pharmacology and Physiology, University of Maryland School of Medicine, Baltimore, MD, USA; 5 Department of Medical Oncology, General University Hospital of Larissa, Larissa, Greece; 6 Laboratory of Rare Human Circulating Cells and Liquid Biopsy (LCCRH), University Medical Centre of Montpellier, Montpellier, France; 7 CREEC, MIVEGEC, University of Montpellier, CNRS, IRD, Montpellier, France; 8 European Liquid Biopsy Society (ELBS), Hamburg, Germany

**Keywords:** Artesunate, circulating tumor cells, anoikis, metastasis, small-cell lung cancer (SCLC), TetherChip, apoptosis

## Abstract

**Objectives**: Circulating tumor cells (CTCs) drive metastasis and exhibit resistance to conventional therapies, making them crucial therapeutic targets. Artesunate (AS), a derivative of artemisinin, displays anticancer activity, including inhibition of JunB proto-oncogene (JUNB) and programmed death ligand-1 (PD-L1) and upregulation of Vimentin (VIM), markers related to poor prognosis in CTCs. This study aimed to evaluate the effects of AS on adherent and non-adherent cancer cell lines (breast, lung, colon), the patient-derived colon cancer CTC-MCC-41 line, and CTCs from small-cell lung cancer (SCLC) patients. **Methods**: AS’s effect was evaluated using TetherChip technology. Cell viability was measured using MTT assay, while immunofluorescence staining and the VyCAP platform were applied to characterize and quantify CTCs. **Results**: AS significantly reduces viability in all tested cell lines in a time- and concentration-dependent manner, with non-adherent cells showing higher resistance. Notably, CTC-MCC-41 cells are the most sensitive to AS treatment. AS demonstrates stronger cytotoxicity than 5-fluorouracil (5-FU) in most cancer models. In SCLC patient samples, AS reduces total CTC counts (*p* < 0.001), eliminates aggressive phenotypes such as (CK+/CXCR4+/JUNB–) and (CK+/VIM+/GLU+), and increases apoptotic (M30+) CTCs (*p* = 0.021). AS additionally impairs structural features like microtentacles, which facilitate CTC reattachment. **Conclusions**: These findings underscore AS’s ability to target metastasis-competent and anoikis-resistant tumor cells, reducing their viability, invasiveness, and survival mechanisms. AS emerges as a promising candidate for anti-metastatic therapy and warrants further investigation in precision oncology.

## 1 Introduction

Circulating tumor cells (CTCs) in the bloodstream and disseminated tumor cells in the bone marrow are key drivers of metastasis [1,2]. The metastatic process involves tumor cell detachment from the primary tumor, survival in circulation, and colonization at distant organs. Since CTCs are precursors to metastasis, targeting them offers a prominent strategy to limit disease progression [3]. However, standard therapies are difficult to combat them, due to their resistance to anoikis, a type of programmed cell death induced by cell detachment, and their ability to evade immune detection [4]. Therefore, a key feature of CTCs is their ability to survive in the absence of extracellular matrix (ECM), which allows them to evade anoikis. To experimentally achieve an anoikis-resistance condition *in vitro*, poly(2-hydroxyethyl methacrylate) (polyHEMA)-coated plates were used to inhibit cell adhesion and induce a suspension state that models anoikis-resistant survival [4]. Recent studies have shown a strong association between anoikis resistance and chemoresistance, as non-adherent cancer cells often display reduced sensitivity to conventional therapies [5]. These findings underscore the need for innovative therapeutic approaches to counteract these survival mechanisms.

Artesunate (AS), a synthetic derivative of artemisinin, has gained recognition for its anticancer properties, with evidence supporting its activity across diverse cancer types [6,7,8,9]. It is also implicated in anoikis-related death [10]. Studies have shown that AS induces cytotoxicity in breast cancer cells, particularly in MCF-7 models [6,7], while also inhibiting key metabolic and survival pathways in non-small cell lung cancer (NSCLC) [8] and promoting reactive oxygen species (ROS)-dependent senescence and autophagy in colorectal cancer [9]. One key mechanism involves the suppression of Activator Protein-1 (AP-1) transcription factors, namely JunB proto-oncogene (JUNB), which regulates genes involved in tumor progression, invasion, and epithelial-mesenchymal transition (EMT) [11,12,13]. JUNB is also known to control the expression of programmed death-ligand 1 (PD-L1), a critical immune checkpoint molecule that enables cancer cells to evade immune detection [14]. Additionally, AS has been reported to influence EMT markers, in particular vimentin (VIM), a cytoskeletal protein linked to enhanced migration and invasion [15].

Notably, our research group has shown, among others, that the expression of JUNB [16,17], PD-L1 [18,19,20], and VIM [21] in CTCs is associated with poor prognosis, in a plethora of cancer types, highlighting their significance as potential therapeutic targets. Additionally, C-X-C chemokine receptor type 4 (CXCR4), a chemokine receptor involved in CTC seeding and metastatic spread [17], has been linked to tumor aggressiveness. Furthermore, detyrosinated α-tubulin (GLU), a microtubule modification known to enhance cellular plasticity and EMT, is highly expressed in CTCs [19,21,22]. Since AS has been shown to modulate these markers [11,15,23], its potential impact on CTC-associated phenotypes remains an interesting subject of ongoing research and warrants further investigation.

Based on this biological and therapeutic context, this study examined the effects of AS on both adherent and non-adherent cancer cell lines derived from breast, lung, and colon cancer. Since non-adherent cells represent an experimental model of anoikis-resistant survival, we also extended our analysis to the first patient-derived colon cancer CTC cell line (CTC-MCC-41) [24] to further validate our findings. To enhance the clinical relevance of this study, we also explored the effects of AS on patient-derived CTCs from small-cell lung cancer (SCLC) patients using TetherChip technology, a high-throughput platform for phenotypic and functional CTC characterization [25], allowing the visualization of microtentacles (McTNs). McTNs are tubulin-based protrusions that facilitate CTC reattachment and metastatic seeding [26]. Overall, the current study aimed to evaluate the impact of AS on the viability of adherent and non-adherent cancer cells and CTCs. It also focused on the expression of prognostic biomarkers (JUNB, CXCR4, VIM, GLU) in these cells before and after AS treatment.

Although AS has been widely investigated in established cancer cell lines, its impact on patient-derived CTCs has not been examined. In this study, we therefore assess the effect of AS using both *in vitro* cancer systems and clinically relevant CTC models, providing insights into AS’s potential to interfere with metastatic progression.

## 2 Materials and Methods

### 2.1 AS Synthesis

AS was synthesized, purified, and structurally characterized by modern spectroscopic techniques, according to a previously reported procedure [27].

All solvents were dried and purified according to standard procedures prior to use. Melting points were determined with a Buchi SMP-20 apparatus (Buchi, Flawil, Switzerland) and are uncorrected. When required, reactions were carried out under an inert atmosphere (dry Ar) in preflamed glassware. Anhydrous Na_2_SO_4_ was used for drying solutions, and the solvents were then routinely removed at ca. 40°C under reduced pressure using a rotary vacuum evaporator. All reagents employed in the present work were commercially available and used without further purification. Flash column chromatography (FCC) was performed on silica gel (70–230 and 230–400 mesh, Merck, Darmstadt, Germany) and analytical thin layer chromatography (TLC) on silica gel 60-F_254_ precoated aluminum foils (0.2 mm film, Merck, KGaA, Darmstadt, Germany). Spots on the TLC plates were visualized with UV light at 254 nm and using *p*-anisaldehyde or charring solution. ^1^H NMR spectra were recorded in CDCl_3_ at 600.13 MHz and ^13^C spectra at 150.9 MHz on a Bruker AVANCEIII HD spectrometer. Chemical shifts (*δ*) are referenced with respect to the residual CHCl_3_ proton of the solvent CDCl_3_ at *δ* = 7.26 ppm. ^13^C NMR spectra were fully decoupled and are referenced to the middle peak of the solvent CDCl_3_ at *δ* = 77.0 ppm. Splitting patterns are designated as: s, singlet; d, doublet; t, triplet; q, quadruplet; dd, double doublet, etc. Coupling constants (*J*) are reported in Hertz. Electrospray Ionization High-Resolution Mass Spectrometry spectra (ESI-HRMS spectra) were recorded at 30 eV on a HESI-ORBITRAP MS, EXPLORIS 120 (Thermo Fischer Scientific, BRE725531, Waltham, MA, USA) using MeOH as the solvent.

To an ice-cooled (0°C) suspension of artemisinin (200 mg, 0.708 mmol) in dry MeOH (1.2 mL), NaBH_4_ (67 mg, 1.77 mmol) was added portion-wise and the reaction mixture was stirred for 2 h at 0°C. Then, the emulsion was neutralized (pH~6) with addition of a solution 30% CH_3_COOH in MeOH, and concentrated under reduced pressure to dryness. The residue was, then extracted twice with EtOAc and the combined organic layers were washed with water and brine, dried over anhydrous Na_2_SO_4_, filtered and concentrated under vacuum. The white residue was subjected to FCC using PhMe/EtOAc 7:3 as eluting system and the afforded intermediate (dihydroartemisinin) was used directly to the next step. Rf (PhMe/EtOAc 7:3) = 0.28, m.p.: 150–151°C.

A solution of succinic anhydride (97.1 mg, 0.969 mmol) in DCM (591 μL) was treated under Ar with 60 μL Et_3_N. To this solution, dihydroartemisinin (197 mg, 0.693 mmol) was added portion-wise, and the resulting mixture was stirred at room temperature for further 6 h. Then, it was quenched with cold aqueous citric acid solution (5%), and the organic layer was washed with water. The aqueous phase was extracted thoroughly with EtOAc, and the combined organic layers were dried over anhydrous Na_2_SO_4_, filtered and concentrated to dryness. The pure product (253 mg, 93% yield) was afforded in the form of white needles after FCC purification using PhMe/EtOAc 8:2 as eluting system. Rf (PhMe/EtOAc 8:2) = 0.27, m.p.: 131–133°C.

^1^H NMR (CDCl_3_, 600 MHz) δ 5.78 (dd, *J* = 9.9, 1.7 Hz, 1 H), 5.43 (d, *J* = 1.38 Hz, 1 H), 2.76–2.69 (m, 3 H), 2.68–2.62 (m, 1 H), 2.55 (tdd, *J* = 12.4, 6.0, 3.7 Hz, 1 H), 2.36 (m, 1 H), 2.02 (dt, *J* = 14.5, 4.1 Hz, 1 H), 1.88 (ddd, *J* = 14.4, 6.6, 3.5 Hz, 1 H), 1.76 (dt, *J* = 12.5, 3.7 Hz, 1 H), 1.71 (m, 1 H), 1.61 (dtd, *J* = 13.9, 4.6, 1.4 Hz, 1 H), 1.48 (ddd, *J* = 13.5, 11.6, 5.0, 1.8 Hz, 1 H), 1.42 (d, *J* = 1.73 Hz, 3 H), 1.39–1.24 (m, 3 H), 1.01 (m, 1 H), 0.95 (dd, *J* = 6.2, 1.5 Hz, 3 H), 0.83 (dd, *J* = 7.2, 1.6 Hz, 3 H);

^13^C NMR (151 MHz) δ 178.0, 171.3, 104.7, 92.5, 91.7, 80.3, 51.8, 45.5, 37.5, 36.4, 34.3, 32.0, 29.2, 28.9, 26.1, 24.8, 22.2, 20.4, 12.2.

HR-HESI-ORBITRAP-MS (30 eV) _m/z_: [M-H]-Calcd for C19H28O8-383.1706, Found: 383.1708.

### 2.2 Cell Culture

MDA-MB-231 (HTB-26, RRID: CVCL_0062), MDA-MB-436 (HTB-130, RRID: CVCL_0623), A549 (CCL-185, RRID: CVCL_0023), H1299 (CRL-5803, RRID: CVCL_0060), and HT-29 (HTB-38, RRID: CVCL_0320) cells were cultured in Dulbecco’s Modified Eagle Medium (DMEM) Glutamax (10566016, Thermo Fisher Scientific, Waltham, MA, USA) which contained 10% fetal bovine serum (FBS) (PAN-Biotech, P40-37500, Aidenbach, Germany), and 50 U/mL penicillin/50 μg/mL streptomycin (15140122, Thermo Fisher Scientific). MCF-7 (HTB-22, RRID: CVCL_0031) cells, were cultured as described above with the addition of 0.01 mg/mL human recombinant insulin (12585014, Thermo Fisher Scientific). DMS 454 (95062832, RRID: CVCL_2438), SW-620 (CCL-227, RRID: CVCL_0547) and CTC-MCC-41 (RRID: CVCL_0I26) cells were cultured in Roswell Park Memorial Institute 1640 Medium (RPMI 1640) (21875034, Thermo Fisher Scientific) containing 1% L-Glutamine and supplemented with 10% FBS, with the latter cell line being also supplemented with 1% insulin, transferrin and selenium (51300044, Thermo Fisher Scientific), epidermal growth factor (EGF; 20 ng/mL) (Qkine, QK011-0100, Cambridge, UK) and fibroblast growth factor (FGF; 10 ng/mL) (QK025-0050, Qkine). CTC-MCC-41 cells were grown in ultra-low attachment flasks (Corning, 3814, New York, NY, USA).

BEAS-2B (CRL-9609, RRID: CVCL_0168) were grown in the designated Bronchial Epithelial Cell Growth Basal Medium (Lonza Bioscience, CC-3171, Walkersville, MD, USA) and their supplements/growth factors, without serum and in flasks/plates lacking any substrate coating [28]. Cells were maintained at 37°C in 5% CO_2_. Cells were sub-cultivated with 0.25% trypsin-EDTA (25200056, Thermo Fisher Scientific).

Colon cancer cell lines and the colon cancer patient-derived CTC-MCC-41 cell line were kindly provided by Professor Alix-Panabières (University Medical Centre of Montpellier, France). The breast MDA-MB-436 cancer cell line was kindly provided by Dr. Martin (University of Maryland School of Medicine, Baltimore, MD, USA). The DMS 454 cell line was obtained from Sigma-Aldrich (Merck KGaA, St. Louis, MO, USA). All other cancer cell lines were obtained from the American Type Culture Collection (ATCC) (Manassas, VA, USA). ATCC’s curated citations support culture conditions used in this study [29,30,31], and numerous peer-reviewed publications from our group report successful growth and experimental use of these cell lines in DMEM or RPMI 1640 media [22,32,33]. The cell lines were authenticated using short tandem repeat (STR) profiling, and all experiments were performed with mycoplasma-free cells.

### 2.3 Prevention of Cell Adherence

To achieve non-adherent conditions, 48-well plates were pre-coated with 10 mg/mL poly(2-hydroxyethyl methacrylate) [polyHEMA] (529257-1G, Sigma-Aldrich) in 95% EtOH, following the manufacturer’s instructions. polyHEMA was left to air-dry overnight in a laminar flow hood and plates were sterilized using the cabinet’s built in Ultraviolet-C (UV-C) germicidal lamp (254 nm) for 20 min before use [5]. In polyHEMA conditions (hydrophilic), cells formed substratum (ECM)-detached 3D spheroids, as previously described for lung, breast, and colon cells [5,34,35]. Spheroid formation was defined by the presence of dense, 3D cellular aggregates with well-defined borders. Integrity and viability were verified by microscopic inspection, where spheroids maintained their spherical morphology without signs of cellular dissociation or debris throughout the experimental period.

### 2.4 Cell Viability

Cells were seeded in 48-well plates coated with (3 × 10^4^ cells/well) or without polyHEMA (15 × 10^3^ cells/well) and were grown for 24 h. Cells were serum-starved for 18 h before the addition of AS (4-oxo-4-[[(1R,4S,5R,8S,9R,10S,12R,13R)-1,5,9-trimethyl-11,14,15,16-tetraoxatetracyclo [10.3.1.04,13.08,13]hexadecan-10-yl]oxy]butanoic acid) and/or 5-fluorouracil (5-FU) (TCI Chemicals, F0151, Tokyo, Japan). Viability was examined by the 3-(4,5-dimethylthiazol-2-yl)-2,5-dimethyltetrazolium bromide (MTT) assay, following 48 h (or 24 h when indicated) treatments [36]. Briefly, 1/10 of the medium’s volume of MTT (5 mg/mL in PBS) was added to each well, resulting in a final concentration of 0.5 mg/mL. Plates were incubated for 2 h at 37°C, protected from light. Formazan crystals were solubilized with acidified isopropanol and the absorbance was measured at 550 nm using a Dynex MRX microplate reader (Dynex Technol. Chantilly, VA, USA). Background absorbance was corrected by subtracting the mean optical density of cell-free (culture medium-containing) wells from all experimental readings before calculating viability.

Full concentration-response analyses were performed in representative cell lines of each cancer type, while additional cell lines were evaluated at selected concentrations of AS (10 μM and 100 μM) and 5-FU (10 μM) to allow comparisons across models. DMSO served as the vehicle for both AS and 5-FU, with final concentrations of 0.1% for 100 μM AS and 0.05% for 10 μM 5-FU. The number of replicates and independent experiments conducted is explicitly mentioned in the text, figure legends, and Table S1.

### 2.5 Patients’ Samples

A total of 5 chemotherapy-naive SCLC patients were enrolled in the study. Patients’ median age was 66 years (range 61–72). The inclusion criteria were; confirmed SCLC diagnosis and no prior anticancer treatment. Exclusion criteria included prior treatment for SCLC, presence of another active malignancy, or severe comorbid conditions. This study adheres to the Declaration of Helsinki guidelines, as updated in 2013, and was approved by the Ethics and Scientific Committees of the University General Hospital of Larissa, 41334 Larissa, Greece (32710/3-8-20). All patients gave their informed written consent for having their blood collected and for their clinical follow-up data to be used for research purposes. Peripheral blood (10 mL), from all patients was collected in EDTA K2 tubes. Peripheral blood mononuclear cells (PBMCs) were isolated from SCLC patients’ blood samples using Ficoll-Hypaque (P04-60500, PAN-Biotech) density gradient centrifugation at 360× *g* for 30 min at room temperature (RT) (NUVE NF 200, Ankara, Turkey). PBMCs were washed twice with PBS and centrifuged at 250× *g* for 10 min at RT. Aliquots of 500,000 cells/500 μL were centrifuged at 450× *g* for 2 min at RT on Superfrost glass slides (22-265446, Thermo Fisher Scientific) using a Hettich Rotofix 32 A centrifuge (Hettich GmbH, Tuttlingen, Germany). After immunofluorescence (IF) staining, one slide from each patient was analyzed for the identification of CTCs and evaluation of the examined molecules, by observation with the VyCAP Puncher system version 1.5.1 (VyCAP B.V., Enschede, The Netherlands). CTC enumeration was performed after IF staining on the cytospin preparations.

### 2.6 CTCs’ Culture and TetherChip Analysis

PBMCs’ fractions (including CTCs) (5 × 10^6^ cells/well) were cultured for 4–5 days, in 6-well plates (SPL Life Sciences, 30006, Pocheon-si, Republic of Korea), in a final volume of 1.5 mL. Cells were cultured in RPMI 1640 (21875034, Thermo Fisher Scientific) containing 1% L-Glutamine and supplemented with 10% FBS (P40-37500, PAN-Biotech), 1% insulin, transferrin and selenium (51300044, Thermo Fisher Scientific), EGF (20 ng/mL) (QK011-0100, QKine) and FGF (10 ng/mL) (QK025-0050, Qkine). The plates were pre-coated with 10 mg/mL polyHEMA as described in [37]. Samples from individual patients were processed independently. Cells were cultured as reported in [37], with medium renewal occurring every two days. Cells capable of surviving under non-adherent conditions, including CTCs, were then characterized with immunofluorescence experiments using the TetherChip platform, a microfluidic device designed to immobilize non-adherent tumor cells on a transparent, cell-repellent surface via lipid-based tethering. This approach permits stable cell retention and compatibility with standard formaldehyde fixation for subsequent high-resolution imaging, as previously reported by Ju et al. (25). Cytokeratin (CK), an epithelial marker, was used to characterize a cell as a CTC. Regarding the (CK/PD-L1/CD45) and (CK/M30/CD45) stainings, the leukocyte marker CD45 was used as a negative marker for CTC selection. In cases where CK expression was low, the cytomorphological criteria, such as high nuclear-cytoplasmic ratio, were also applied for CTCs’ identification, as described in [17]. The identification of CTCs was performed blindly to clinical data. In contrast, most of the PBMCs in this distinct cell culture medium and conditions after five days of culture did not survive.

Before AS treatment, cells were incubated in serum-free medium for 24 h to deplete serum factors. Subsequently, cells were treated with 10 μM AS in serum-free medium for 24 h, while cells treated with 10 μM DMSO (AS carrier) served as controls. This concentration was kept constant across all experimental groups. Following treatment, cells were placed in different wells of the Tetherchip containing 100,000 cells per well and allowed to tether for 45 min. All experiments were performed in triplicate.

### 2.7 Immunofluorescence on TetherChips

IF stainings of CK (1:70 in PBS/1% FBS, 1 h, RT) (Amgen, Thousand Oaks, CA, USA) (RRID: AB_11218704)/PD-L1 (1:100 in PBS/1% FBS, 1 h, RT) (Novus Biologicals, NB300-903, Littleton, CA, USA) (RRID: AB_10986627)/CD45 (1:100 in PBS/1% FBS, 1 h, RT) (Santa Cruz Biotechnology, sc-1178 AF647, Dallas, TX, USA) (RRID: AB_627074), CK/VIM (1:100 in PBS/1% FBS, 1 h, RT) (Santa Cruz Biotechnology, sc-6260) (RRID: AB_628437)/GLU (1:100 in PBS/1% FBS, 1 h, RT) (Abcam, ab48389, Cambridge, MA, USA) (RRID: AB_869990), and CK/M30 (1:100 in PBS/1% FBS, 1 h, RT) (Roche Diagnostics GmbH, 12 140 322 001, Mannheim, Germany) (RRID: AB_1613872)/CD45 were performed according to protocols described in [37], while the CK/CXCR4 (1:100 in PBS/1% FBS, 1 h, RT) (Abcam, ab124824) (RRID: AB_10975635)/JUNB (1:100 in PBS/1% FBS, 1 h, RT) (Santa Cruz Biotechnology, sc-46) (RRID: AB_2130002) staining was conducted as detailed in [17].

IF staining of Wheat germ agglutinin (WGA) (1:100 in PBS/1% FBS, 1 h, RT) (Thermo Fisher Scientific, W11261)/CD45 was also performed to visualize CTCs’ McTNs as in [37]. The current study focused on evaluating AS’s effect on CTC viability and expression of prognostic biomarkers [JUNB (Santa Cruz Biotechnology, sc-46), CXCR4 (Abcam, ab124824), VIM (Santa Cruz Biotechnology, sc-6260), GLU (Abcam, ab48389)].

Regarding all IF staining panels, fixation was carried out using 3.7% formaldehyde in PBS for 10 min, followed by permeabilization with 0.1% Triton-X 100 in PBS for 10 min at RT. Non-specific binding was blocked by incubating the cells in 5% FBS in PBS for 1 h at RT. For the observation of McTNs, WGA conjugated to Alexa Fluor 488 (1:100 dilution in PBS/1% FBS, Thermo Fisher Scientific, W11261, 45 min, RT) was used, as WGA binds to mammalian cell membranes, highlighting the presence of McTNs [38]. After three washes with PBS, CD45 conjugated to Alexa Fluor 647 (sc-1178, Santa Cruz Biotechnology) was added to verify whether cells with McTNs were CTCs. For nuclear staining, Hoechst nuclear stain (Cell Signaling Technology, 4082S, Danvers, MA, USA) (1:2500 dilution in PBS/1% FBS, 15 min, RT) was used. Visualization of the stained cells was performed using the VyCAP Puncher system version 1.5.1 (VyCAP B.V.).

### 2.8 Statistical Analysis

All results are expressed as mean ± SEM unless otherwise specified. For experiments with *n* = 2, data are presented as the mean of two independent experiments, and error bars represent the range. Cell viability was assessed and reported as viability (%) or decrease in viability (%), normalized to control conditions. Depending on the experiment, the control was either 0.1% DMSO-treated cells (for concentration-response experiments) or untreated cells (for single-dose comparisons). Statistical analyses were performed using GraphPad Prism (version 8, La Jolla, CA, USA). Concentration-response relationships were fitted using nonlinear regression models. IC_50_ values were converted to pIC_50_, where values are expressed on a scale that better approximates normality. Paired samples non-parametric tests or paired samples *t*-tests were applied where appropriate. A *p*-value of <0.05 was considered statistically significant. For *n* = 2, due to limited sample size, statistical significance was not calculated; however, the range indicates the consistency of the observed effect, and results were interpreted descriptively.

## 3 Results

### 3.1 Concentration-Dependent Effect of AS on the Viability of Lung, Breast, and Colon Cancer Cells

**Figure 1:**
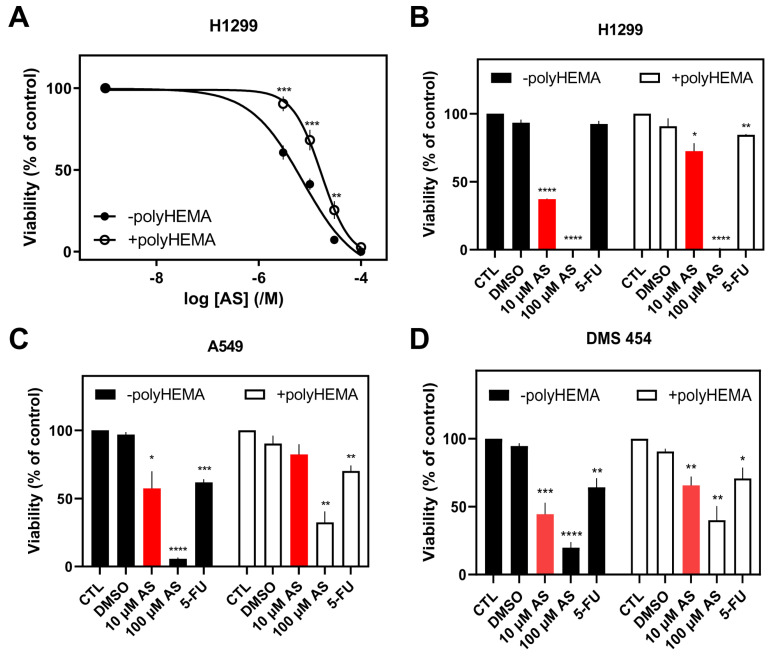
Effect of Artesunate (AS) on lung cancer cells’ viability. (**A**,**B**) H1299, (**C**) A549, and (**D**) DMS 454 cells (adherent or not) were treated with different AS concentrations (10 μM & 100 μM) (**A**–**D**) and 5-fluorouracil (5-FU) (10 μM; **B**–**D**) for 48 h. Viability (%) is normalized against carrier-treated (**A**; 0.1% DMSO) or untreated cells (**B**–**D**). Results are expressed as mean ± Standard Error of the Mean (SEM) from independent experiments performed in duplicates, where **A** (*n* = 10), **B** (*n* = 4), **C** (*n* = 4), and **D** (*n* = 3). *p < 0.05, **p < 0.01, ***p < 0.001, ****p < 0.0001.

**Figure 2:**
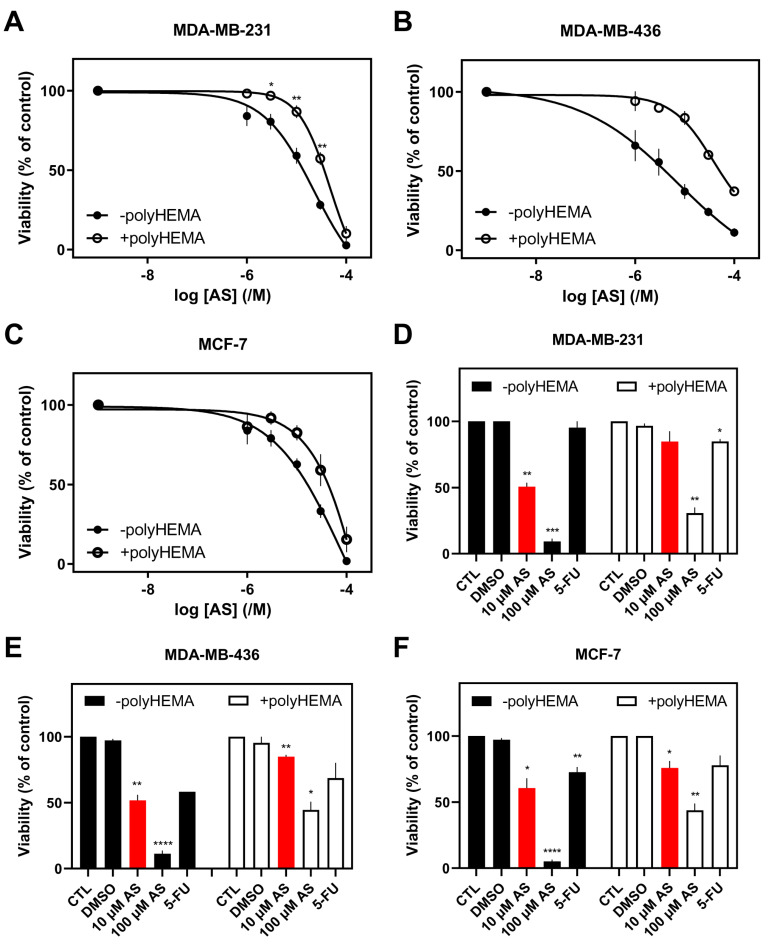
Effect of AS on breast cancer cells’ viability. (**A**,**D**) MDA-MB-231, (**B**,**E**) MDA-MB-436, and (**C**,**F**) MCF-7 cells (adherent or not) were treated with different AS concentrations (10 μM & 100 μM) (**A**–**F**) and 5-FU (10 μM; **D**–**F**) for 48 h. Viability (%) is normalized against carrier-treated (**A**–**C**; 0.1% DMSO) or untreated cells (**D**–**F**). Results are expressed as mean ± SEM (or mean ± range for *n* = 2) from independent experiments performed in duplicate, where **A** (*n* = 10), **B** (–polyHEMA *n* = 4; +polyHEMA *n* = 2), **C** (*n* = 4), **D** (*n* = 3), **E** (–polyHEMA *n* = 4; +polyHEMA *n* = 3; 5-FU *n* = 2), and **F** (*n* = 4). *p < 0.05, **p < 0.01, ***p < 0.001, ****p < 0.0001.

**Figure 3:**
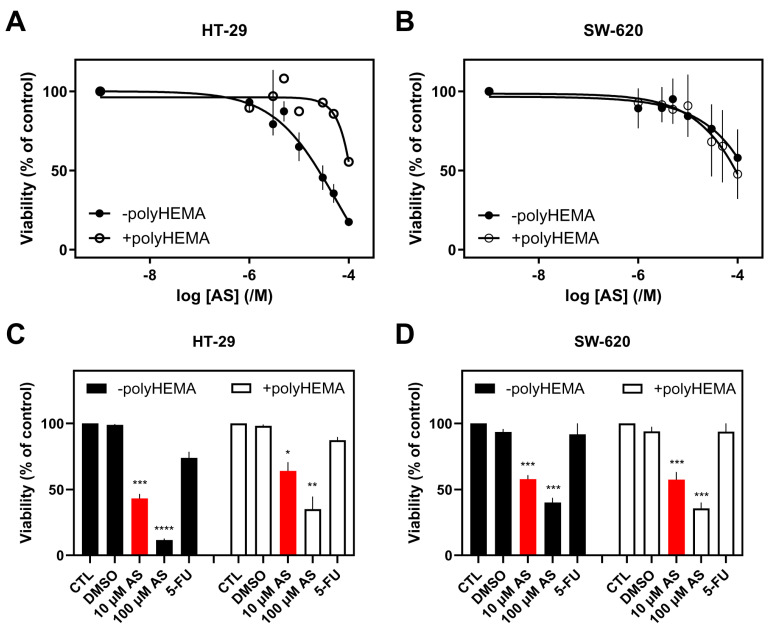
Effect of AS on colon cancer cells’ viability. (**A**,**C**) HT-29, and (**B**,**D**) SW-620 cells (adherent or not) were treated with different AS concentrations (10 μM & 100 μM) (**A**–**D**) and 5-FU (10 μM; **C**,**D**) for 48 h. Viability (%) is normalized against carrier-treated (**A**,**B**; 0.1% DMSO) or untreated cells (**C**,**D**). Results are expressed as mean ± SEM (or mean ± range for *n* = 2) from independent experiments performed in duplicate, where **A** (–polyHEMA *n* = 4; +polyHEMA *n* = 2), **B** (± polyHEMA *n* = 2), **C** (*n* = 4; 5-FU *n* = 2), and **D** (*n* = 4; 5-FU *n* = 2). *p < 0.05, **p < 0.01, ***p < 0.001, ****p < 0.0001.

To examine the effect of AS on the viability of various cell lines from different tissues, cells were treated with a range of concentrations of AS for 48 h in serum-free conditions. Results on viability (%) were normalized against carrier-treated cells (0.1% DMSO corresponding to 100 μM AS; control). IC_50_ (concentration at which 50% inhibition was observed) and pIC_50_ (−logIC_50_) values for the different cell lines were calculated. The MTT assay was used to determine viable cells. AS was found to reduce cell viability in a concentration-dependent way in all cancer cell lines, irrespective of their tissue origin (Fig. 1, Fig. 2 and Fig. 3; closed circles).

Regarding the lung cancer cell line H1299, IC_50_ (pIC_50_) values of 9.30 ± 1.74 μM (5.11 ± 0.10, *n* = 10) were calculated (Fig. 1A; closed circles). In breast cancer cell lines, IC_50_ (pIC_50_) values of 19.3 ± 2.34 μM (4.75 ± 0.064, *n* = 10), 13.6 ± 6.00 μM (5.00 ± 0.20, *n* = 4), and 34.6 ± 5.00 μM (4.47 ± 0.062, *n* = 4) were calculated for MDA-MB-231 (Fig. 2A), MDA-MB-436 (Fig. 2B), and MCF-7 (Fig. 2C), respectively. MCF-7 had statistically higher IC_50_ compared to MDA-MB-231 and MDA-MB-436 (*p* < 0.01 and *p* < 0.05, respectively) (Fig. 2A–C; closed circles).

Finally, in colon cancer cell lines, IC_50_ (pIC_50_) values of 39.0 ± 17.4 μM (4.58 ± 0.24, *n* = 4) and 45.9 ± 21.3 μM (4.35 ± 0.21, *n* = 2) were calculated for HT-29 (Fig. 3A) and SW-620 (Fig. 3B) cells, respectively (Fig. 3; closed circles).

**Figure 4:**
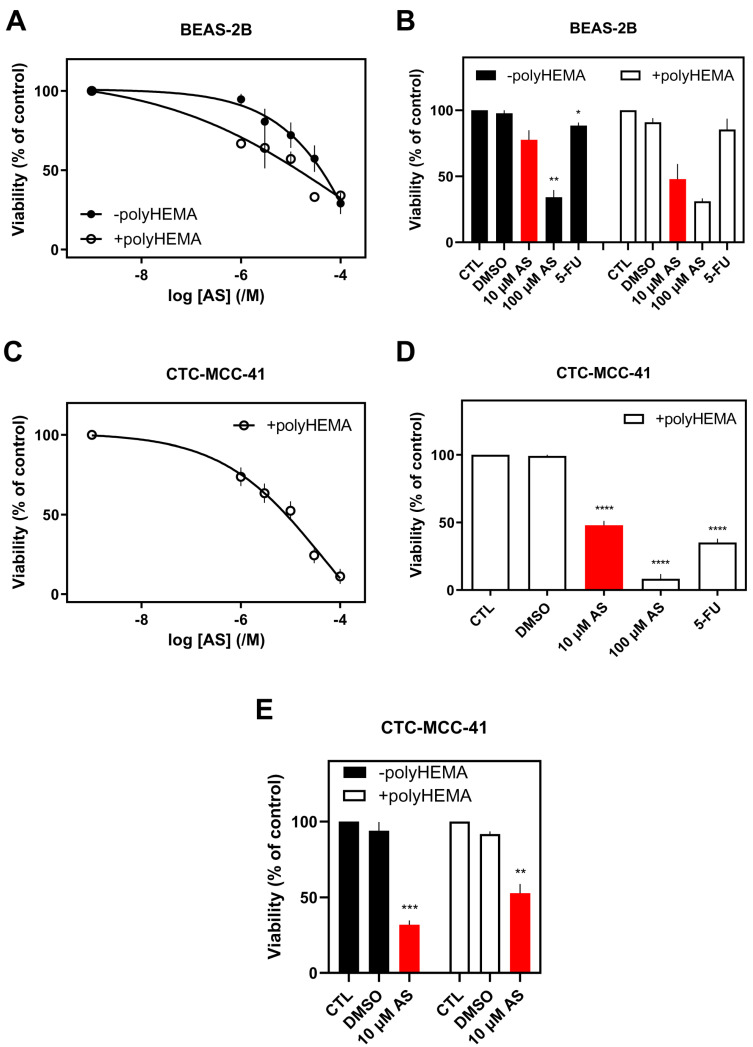
Effect of AS on normal epithelial BEAS-2B and the patient-derived colon Circulating tumor cell (CTC) cell line, CTC-MCC-41 cells’ viability. Cells (adherent or not) were treated with different AS concentrations (10 μM & 100 μM) (**A**–**E**) and 5-FU (10 μM; **D**) for 48 h. Viability (%) is normalized against carrier-treated (**A**,**C**; 0.1% DMSO) or untreated cells (**B**,**D**,**E**). Results are expressed as mean ± SEM (or mean ± range for *n* = 2) from independent experiments performed in duplicate, where **A** (–polyHEMA *n* = 5; +polyHEMA *n* = 2), **B** (–polyHEMA *n* = 4; +polyHEMA *n* = 2), **C** (*n* = 5), **D** (*n* = 7), and **E** (*n* = 4). *p < 0.05, **p < 0.01, ***p < 0.001, ****p < 0.0001.

Based on the origin of the cells, lung cancer cells appeared to have lower IC_50_, followed by breast and colon cancer cells, suggesting an increased sensitivity of these cells, respectively. AS was also found to dose-dependently reduce the viability of non-tumorigenic, normal human bronchial epithelial cells, BEAS-2B (Fig. 4A; closed circles). BEAS-2B cells, however, were less sensitive to AS with an IC_50_ value of 102 ± 37.8 μM (pIC_50_ = 4.11 ± 0.16, *n* = 5) at 48 h.

Selectivity indices were calculated by dividing IC_50_ values for cytotoxicity against normal BEAS-2B cells with the IC_50_ values for cytotoxicity against the lung cancer cell line H1299, showing 11-fold lesser toxicity of AS in normal cells compared to H1299 cells.

Overall, irrespective of tissue origin, cells can be ranked in terms of their sensitivity to AS (based on the IC_50_ values) with H1299 being more sensitive, followed by MDA-MB-436, MDA-MB-231, HT-29, MCF-7, SW-620, and BEAS-2B, as the least sensitive cells to AS. Interestingly, this sensitivity order correlates with the aggressiveness of cancer cell lines, with higher sensitivity observed in more aggressive models such as H1299, MDA-MB-436 and MDA-MB-231, and lower sensitivity in less aggressive, like MCF-7, HT-29, and BEAS-2B. This implies that AS could be more effective in aggressive tumor types.

### 3.2 Concentration-Dependent Effect of AS on the Viability of Non-Adherent Lung, Breast, and Colon Cancer Cells

To examine the effect of AS on the viability of the same cancer cell lines after preventing their adherence, cells were grown in polyHEMA-coated wells and treated with the same range of concentrations for 48 h in serum-free conditions. This setup was chosen to resemble CTCs’ non-adherent conditions in the bloodstream. AS reduced cell viability in a concentration-dependent manner in all cells (Fig. 1, Fig. 2 and Fig. 3; open circles). A rightward shift of the curve was observed in lung H1299 (Fig. 1A) and breast cancer cell lines (Fig. 2A–C), indicative of an increase in IC_50_ values in the non-adherent set-up.

Regarding the H1299 lung cancer cell line, IC_50_ (pIC_50_) values of 27.0 ± 7.86 μM (4.72 ± 0.12, *n* = 10) were calculated in the non-adherent condition vs. 9.30 ± 1.74 in adherent cells (Fig. 1A). In breast cancer cell lines, IC_50_ (pIC_50_) values of 40.0 ± 8.00 μM (4.46 ± 0.074, *n* = 10), 22.7 ± 19.1 μM (4.69 ± 0.39, *n* = 2), and 45.3 ± 10.6 μM (4.39 ± 0.12, *n* = 4) were calculated for MDA-MB-231 (Fig. 2A), MDA-MB-436 (Fig. 2B), and MCF-7 (Fig. 2C), respectively, while the corresponding IC_50_s as previously described in adherent conditions were 19.3 ± 2.34, 13.6 ± 6.00 and 34.6 ± 5.00, respectively. MCF-7 again had a higher IC_50_ compared to MDA-MB-231 and MDA-MB-436, but not statistically significant. Especially for H1299 and MDA-MB-231, the increase of IC_50_ values in the non-adherent set-up was statistically significant (*p* < 0.05) compared to the values of the adherent cells.

Finally, in colon cancer cell lines, IC_50_ (pIC_50_) values of 5.38 ± 3.17 μM (5.29 ± 0.26, *n* = 2) and 72.3 ± 77.7 μM (4.22 ± 0.52, *n* = 2) were calculated for HT-29 (Fig. 3A) and SW-620 (Fig. 3B) cells, respectively.

In the BEAS-2B cell line, prevention of attachment decreased IC_50_ to 6.19 ± 9.14 μM (pIC_50_ = 5.38 ± 0.82, *n* = 2) (Fig. 4A), suggesting that normal cells are very sensitive in free-floating conditions and hence more susceptible to AS.

Overall, irrespective of tissue origin, cells can be ranked in terms of their sensitivity (based on the IC_50_ values) to AS in non-adherent conditions as follows: BEAS-2B, HT-29, H1299, MDA-MB-436, MDA-MB-231, MCF-7, SW-620, as the least sensitive cells to AS.

### 3.3 Effect of AS and 5-FU on the Viability of Adherent and Non-Adherent Lung, Breast, and Colon Cancer Cells

Subsequently, the effect of AS on adherent and non-adherent cells was compared to a common chemotherapeutic agent, 5-FU. As a more biologically prominent concentration, 10 μM of AS (approximately the average of IC_50_s for the more AS-sensitive H1299, MDA-MB-231, and MDA-MB-436) was chosen for the remaining experiments. The same concentration was chosen for 5-FU to maintain experimental consistency and more direct comparisons between the two agents. Interestingly, all the adherent and the majority of non-adherent cell lines were less sensitive to 5-FU than AS.

Analytically, the most pronounced difference in toxicity (decrease of viability), following treatment with 10 μM AS, between adherent and non-adherent cells, was observed in H1299 (35%), followed by MDA-MB-231 (34%), MDA-MB-436 (33%), A549 (25%), DMS 454 (22%) HT-29 (21%), and MCF-7 (16%) cells (Fig. 1B–D, Fig. 2D–F, Fig. 3C and Table S1). This order correlates with the cells’ aggressiveness for the respective types of cancer, implying that less aggressive cell lines are not influenced by the adherent or non-adherent status of the cells in response to AS. Indeed, in terms of the lung, H1299 (more aggressive cells), showed the highest difference in viability between adherent vs. non-adherent conditions compared to the lesser effect observed in A549 and DMS 454. Similarly, MCF-7, the most “benign” of breast cancer cells, showed reduced sensitivity to AS and a reduced difference in toxicity compared to the other breast cancer cell lines such as MDA-MB-231 and MDA-MB-436.

Interestingly, the effect of 10 μM AS on normal lung cancer cells, BEAS-2B, was smaller (22 ± 7% inhibition of viability) compared to all cancer cell lines in adherent conditions (Fig. 4B and Table S1). In addition, as was the case with the IC_50_ data, the effect of AS on non-adherent normal cells was more pronounced probably due to the vulnerability of normal cells when grown without focal adhesion contacts to the substrate or neighboring cells (53 ± 16% reduction of viability, Fig. 4B and Table S1).

Higher concentration of AS (100 μM, 48 h) was very toxic for most adherent cells (80–100%, except for SW-620, with 60% inhibition of viability) and non-adherent (55%–99%) cell lines (Table S1). 100 μM AS on adherent BEAS-2B decreased viability by approximately 66% and by 69% on non-adherent cells (Table S1), agreeing with the results obtained with 10 μM AS treatment.

5-FU (10 μM, 48 h) had less pronounced effect on the viability of H1299, MDA-MB-231, and SW-620 cells in both adherent and non-adherent conditions (Table S1). 5-FU was more effective in reducing viability of A549, DMS 454, MDA-MB-436, MCF-7 and HT-29 adherent cells (Table S1). In these cancer cells, culturing under non-adherent conditions, followed the pattern observed in AS, leading to a smaller inhibition of viability (not statistically significant).

Time dependence (24 h vs. 48 h) of the effect of AS (10 and 100 μM) and 5-FU for some cells is presented in Fig. S1, whereby longer treatment (48 h) had more effect than 24 h and 5-FU was again less effective than AS in the majority of the cancer cell lines.

### 3.4 Effect of AS and 5-FU on the Viability of a Patient-Derived Colon CTC Line

To investigate the impact of AS on real metastasis-driven CTCs, we evaluated the effect of AS on CTCs from the first patient-derived colon CTC line, CTC-MCC-41 [24]. CTC-MCC-41 were normally grown in suspension, and AS was found to inhibit their viability in a concentration-dependent way with an IC_50_ of 54.6 ± 23.9 μM (*n* = 5; Fig. 4C).

Interestingly, the effect of AS on the CTC line was more prominent than on all the other malignant cell lines, both in adherent and non-adherent status. Namely, treatment for 48 h with 10 μM of AS decreased the viability of adherent cells by 68 ± 3%. This effect was more modest in non-adherent cells with 48 ± 3% inhibition of viability, which was still higher compared to all other cancer cell lines under similar conditions (Fig. 4E and Table S1) and more evident after 48 h of treatment (Fig. S1). 100 μM of AS inhibited the viability of floating colon CTC line by 92 ± 3%.

### 3.5 Effect of AS on CTCs Isolated from SCLC Patients and TetherChip Analysis

Subsequently, to confirm previous results, this study assessed the effect of AS on patient-derived CTCs. PBMCs from five SCLC patients were isolated using Ficoll density gradient centrifugation, cultured for 4–5 days (Fig. S2A), and analyzed with TetherChip technology. A representative cell culture photo from an SCLC patient, after five days of culture is provided in Fig. S2A. We have previously demonstrated that this protocol enables the evaluation of drug efficacy on patient CTCs, offering valuable insights into anti-metastatic agents that can limit the dissemination of tumor cells [37].

**Figure 5:**
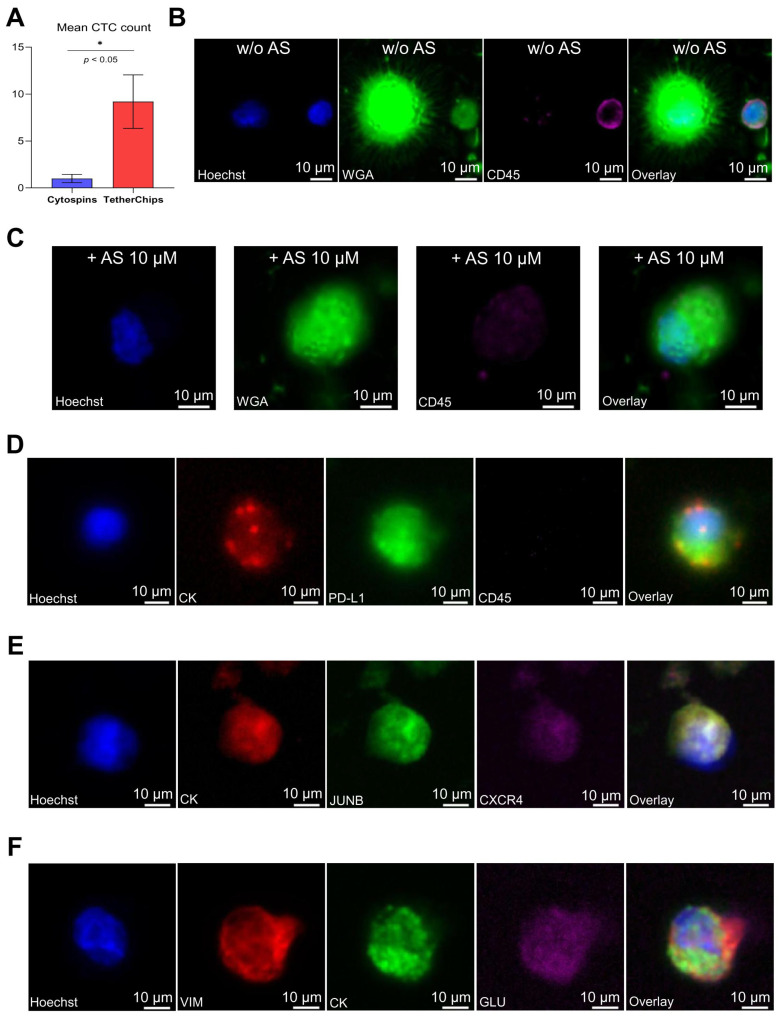
Phenotypic characterization of patient-derived Small-Cell Lung Cancer (SCLC) CTCs. (**A**) Mean number of CTCs detected in cytospins and TetherChips. (**B**) Microtentacles (McTNs) in CTCs detected in PBMCs fraction after culture, without AS treatment, and appearance of McTNs. Nuclei are stained with blue (Hoechst), the cell membrane is stained with green (Wheat germ agglutinin, WGA), while the peripheral blood mononuclear cells (PBMCs) are stained with purple [cluster of differentiation (CD45). The image shows a CTC with McTNs and a PBMC. (**C**) PBMCs fraction (including CTCs) after treatment with 10 μM AS for 24 h. Nuclei are stained with blue (Hoechst), the cell membrane is stained with green (WGA), while PBMCs are stained with purple (CD45). The image shows a CTC without McTNs. (**D**) Immunofluorescence (IF) staining of a CTC for [Cytokeratin (CK)/Programmed death-ligand 1(PD-L1)/CD45] before AS treatment. Hoechst staining of cell nuclei is shown in blue, CK in red, PD-L1 in green, and CD45 in purple. (**E**) IF staining of a CTC for [CK/C-X-C motif chemokine receptor 4 (CXCR4)/JunB proto-oncogene (JUNB)] before AS treatment. Hoechst staining of cell nuclei is shown in blue, CK in red, JUNB in green, and CXCR4 in purple. (**F**) IF staining of a CTC for [CK/vimentin (VIM)/detyrosinated α-tubulin (GLU)] before AS treatment. Hoechst staining of cell nuclei is shown in blue, CK in green, VIM in red, and GLU in purple. Scale bars represent 10 μm. *p < 0.05.

The initial number of CTCs detected after blood collection (500,000 PBMCs per patient in cytospins) was compared to the number of CTCs after cell cultures and analysis in TetherChips. The detection of CTCs after cell culture was significantly increased by 800% compared to cytospins (*p* = 0.02; Fig. 5A). Consequently, this protocol provided high enrichment of CTCs per patient, offering adequate CTC numbers to evaluate drug efficacy. The number of isolated CTCs per patient is shown in Table S2.

McTN formation, which facilitates metastatic dissemination, was assessed in CTCs from SCLC patients using WGA staining before and after AS treatment (Fig. 5B,C). Particularly, IF staining (WGA/CD45) was performed on TetherChips to analyze the presence of McTNs in CTCs. CD45 was added to verify that cells with McTNs were indeed CTCs, as CD45 is a hematopoietic marker and it is absent in CTCs.

Distinct phenotypes (CK+/PD-L1/CD45− and CK+/CXCR4/JUNB) related to poor prognosis were also evaluated in CTCs before and after AS treatment. Fig. 5D–F shows representative images of different phenotypes after culture before AS treatment. The intracellular distribution of these different molecules did not change after AS treatment (data not shown); however, the total number of CTCs expressing these molecules was different.

Furthermore, it has been shown that McTNs are supported by GLU and VIM [39]. Therefore, IF staining (CK/VIM/GLU) in CTCs was also performed before and after AS treatment to further investigate its effects on CTC structural dynamics (Fig. 5F).

In all experiments, control samples with 0.1% DMSO were used for normalization.

**Figure 6:**
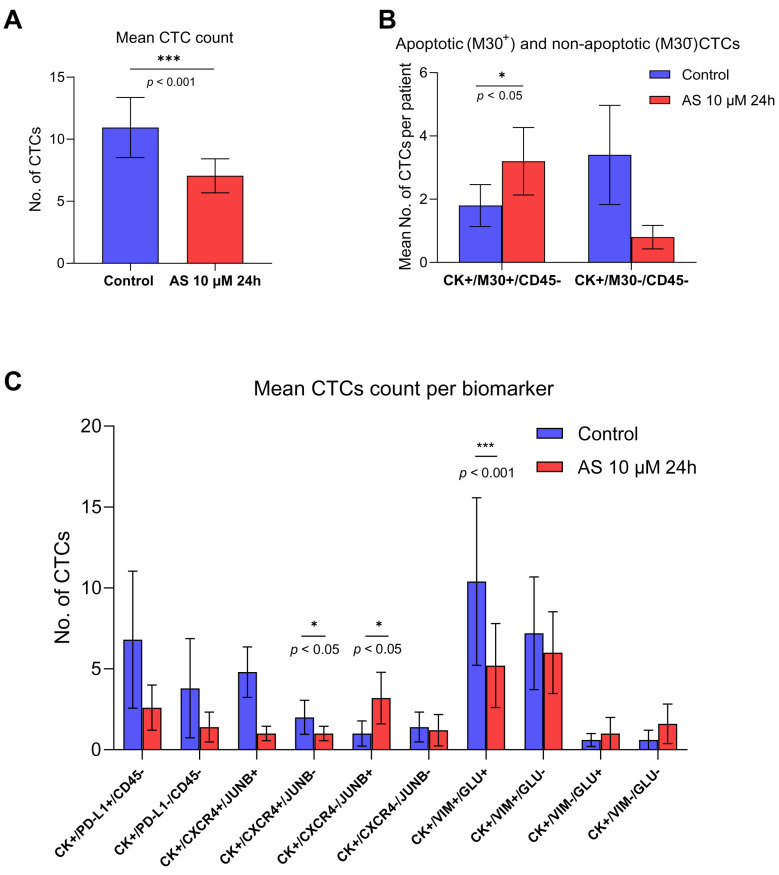
Effects of Artesunate (AS) on Circulating Tumor Cell (CTC) viability and phenotypes in Small-Cell Lung Cancer (SCLC) patient-derived samples. (**A**) Mean number of CTCs detected in TetherChips before and after AS treatment. (**B**) Comparison of apoptotic CTCs before and after AS treatment. Bars represent mean CTC counts per patient (**C**) Distribution of CTC subpopulations based on immunofluorescence staining panels in untreated (control, CTL) and AS-treated conditions. *p < 0.05, ***p < 0.001.

Particularly, quantitative analysis of the total detectable CTCs after treatment with AS for 24 h revealed a reduction of 64% compared to the total number of CTCs in the control samples (*p* = 0.001), demonstrating AS’s cytotoxic effect on patient-derived CTCs (Fig. 6A). The sum of the total CTC numbers from the 4 different IF staining experiments per patient (CK/PD-L1/CD45, CK/JUNB/CXCR4, CK/VIM/GLU, and CK/M30/CD45) is shown in Table S3.

To further evaluate the apoptotic effects of AS on patient-derived CTCs, IF staining with M30 (apoptotic marker)/CD45 antibodies was performed (Fig. S2B,C).

The number of (CK+/M30+/CD45–) apoptotic CTCs was significantly higher in AS-treated samples compared to controls (*p* = 0.021) (Fig. 6B, Table S4), while (CK+/M30–/CD45–) non-apoptotic CTCs were also reduced after AS treatment, but this difference was not statistically significant (Fig. 6B, Table S4). The increase in apoptotic CTCs and the simultaneous reduction in non-apoptotic CTCs following AS treatment indicate that AS could promote apoptotic cell death in SCLC patient-derived CTCs (Fig. 6B).

However, the total number of CTCs harboring McTNs on their surface before and after AS treatment was reduced but not in a statistically significant manner (Table S5, *p* = 0.250). In addition, quantitative analysis of CTC phenotypes, based on the previously described IF panels, before and after AS incubation, revealed significant reductions of specific CTC-subclones (Fig. 6C, Table S6). Representative images of the CTC phenotypes depicted in Fig. 6C are presented in Figs. S3–S5, regarding the CK/PD-L1/CD45 (Fig. S3), CK/CXCR4/JUNB (Fig. S4), and CK/VIM/GLU (Fig. S5) stainings, respectively. Particularly, CTCs expressing the (CK+/CXCR4+/JUNB–) and (CK+/VIM+/GLU+) CTCs were significantly reduced compared to control samples (Fig. 6C, Table S6), suggesting that AS targets aggressive CTC subpopulations linked to metastatic progression.

## 4 Discussion

Anoikis resistance is a crucial feature of metastatic cancer cells, enabling them to evade cell death, resist chemotherapeutics, and enhance their metastatic potential [5,40,41]. This trait is particularly relevant for CTCs, which must survive in circulation to initiate metastasis. Anoikis resistance has also been linked to reduced sensitivity to conventional therapies, complicating treatment strategies [5,40,41].

Artemisinin derivatives, including AS, exhibit antitumor activity by targeting multiple oncogenic pathways, notably those involved in metastasis suppression [8]. AS has been shown to inhibit colon cancer cell growth via ROS-mediated senescence and autophagy [9], a process that may enhance its efficacy against cancer stem cells, which often evade senescence [42]. Additionally, AS has been linked to EMT inhibition [15] and immune modulation through immune checkpoint regulation [43], further supporting its potential as a therapeutic agent. Beyond these effects, recent *in vivo* studies have shown that AS can significantly modulate cellular glucose and lipid metabolism, as shown in db/db mouse models using metabolomic and transcriptomic approaches [44]. Notably, clinical metabolomics analyses have revealed that dysregulation of metabolic pathways is an important feature of lung cancer patients [45], highlighting the relevance of metabolism regulation to lung cancer progression and therapeutic response. Based on this context, AS-associated phenotypic effects observed in this study could potentially relate to the disruption of metabolic pathways that support tumor cell survival.

This study comprehensively evaluated the effects of AS across multiple cancer models, including adherent and anoikis-resistant cell lines, as well as SCLC patient-derived CTCs. Viability assays, TetherChip analysis, and IF staining were employed to assess AS-induced cytotoxicity, phenotypic changes, and apoptosis. In this study, we compared treated vs. untreated CTCs in short-term cultures that do not affect the expression of important prognostic biomarkers in these cells [37]. It is critical to find specific drugs to combat metastatic dissemination; therefore, short-term culture of CTCs is the only way to test the effect of these drugs in real samples, not in cell lines.

Under adherent conditions, AS exhibited stronger cytotoxic effects in aggressive cancer cell lines, with the highest sensitivity observed in H1299, MDA-MB-436, and MDA-MB-231 cells (Fig. 1 and Fig. 2 and Table S1). These findings align with previous reports demonstrating AS’s concentration-dependent inhibition of lung [46,47,48], breast [6,7,49,50], and colon [51] cancer cell viability. Notably, AS showed minimal cytotoxicity in BEAS-2B non-tumorigenic epithelial cells (Fig. 4 and Table S1), reinforcing its potential as a selective anticancer agent. The observed variability in AS sensitivity across the studied cell models could reflect biological heterogeneity, indicating that AS’s effects are model dependent and should be interpreted based on specific cellular context rather than attributed only to tumor origin.

To assess AS’s effects on non-adherent cancer cells, polyHEMA-coated plates were used to prevent cell attachment, thereby modeling anoikis-resistant survival associated with CTCs [5]. AS significantly reduced the viability of H1299, HT-29, DMS 454 and SW-620 cells, (Table S1), underscoring AS’s potential as a promising therapeutic strategy for non-adherent and disseminated tumor cells, mainly for colon and lung cancer patients. These findings indicate that AS exerts its cytotoxic activity even under non-adherent conditions, which resemble anoikis-resistant survival. The scale of this effect varies across cancer cell lines, suggesting that anoikis resistance is insufficient in predicting AS sensitivity. The observed variability implies that AS responses cannot be attributed to a single defining feature, highlighting the complexity of drug sensitivity across cancer cell models.

At higher concentrations (100 μM), AS-induced cytotoxicity was enhanced across all cell lines, indicating significant efficacy in suspension conditions (Table S1).

5-FU is a widely used chemotherapy drug that primarily targets rapidly proliferating cancer cells by inhibiting thymidylate synthase, disrupting DNA replication, and leading to cell cycle arrest [52]. It is commonly used to treat various solid tumors, such as colorectal [53], breast [54], and lung [55] cancers. AS (10 μM) demonstrated superior cytotoxicity compared to 5-FU (10 μM) in both adherent and non-adherent conditions in most cancer cell lines (Fig. 1, Fig. 2, Fig. 3 and Fig. 4 and Table S1), reinforcing the potential importance of AS for cancer treatment.

Additionally, AS significantly reduced the viability of CTC-MCC-41, the first patient-derived colon cancer CTC line [24] (Fig. 4C). These cells exhibited the highest sensitivity to AS compared to all the examined cancer cell lines, with a marked decrease in viability under both adherent and non-adherent conditions (Fig. 4C–E and Table S1). The use of a patient-derived CTC model, such as the CTC-MCC-41 cell line, is particularly valuable, as it represents the biological behavior of metastatic disease. Unlike traditional cancer cell lines that may acquire genetic and phenotypic alterations and represent more contrived cancer models [56], CTC-MCC-41 retains critical CTC characteristics, including epithelial-mesenchymal plasticity and anoikis resistance [31]. These traits are key contributors to metastatic progression, emphasizing the relevance of patient-derived CTCs in evaluating anti-metastatic therapies. AS’s ability to effectively target these aggressive, suspension-surviving cells further underscores its potential as a promising therapeutic strategy against metastatic disease. It should be noted that long-term cultured CTC-derived cell lines, like CTC-MCC-41, may not completely encapsulate the heterogeneity of patient-derived CTCs, and therefore act as a different model, not a direct representation of *in vivo* CTC behavior.

Given the critical role of CTCs in metastasis and their resistance to conventional therapies, further investigation was conducted to assess AS’s effects on patient-derived CTCs. To achieve this, CTCs isolated from five SCLC patients were analyzed using TetherChip technology and IF staining to evaluate AS’s impact on CTC viability, apoptosis, and phenotypic alterations. TetherChip technology offers a useful platform for phenotypic and functional CTC characterization, enabling evaluation of viable cells, observation of McTNs, and real-time assessment of treatment response [25]. Compared to direct cytospin-based evaluation of CTCs, which are prone to significant cell loss, thereby underestimating CTC counts, cell culture and TetherChip technology enhance CTC recovery by preserving cell integrity. Previous reports demonstrated cytospin-related CTC loss rates of 37–51% [57] and reduced detection sensitivity due to preparation-related losses [58]. By minimizing cell loss and maintaining cell viability, our protocol enables improved assessment of metastatic potential and drug response.

Comparison of identified CTCs between cytospins and TetherChips (after 4–5 days of culture) revealed an 800% enrichment of CTCs in TetherChips (*p* = 0.02) (Fig. 5A), reinforcing the protocol’s efficacy in enhancing CTC recovery. This is particularly crucial for drug sensitivity testing, as CTCs are typically present in low numbers in the bloodstream, making functional characterization challenging [59].

IF staining was used to assess CTC subpopulations following AS treatment. Although AS’s anticancer properties are well-documented [60], its effects on real patient-derived CTCs remain unexplored. Given the aggressive nature of SCLC, it was deemed interesting to evaluate CTCs from these patients and to examine established subclones associated with tumor progression, immune evasion, and treatment resistance. McTN formation was also evaluated, as these thin protrusions are linked to tumor cell plasticity and metastatic potential [26]. Consistent with previous findings in triple-negative breast cancer (TNBC) patient-derived CTCs [37], McTNs were detected in cultured SCLC patients’ CTCs before AS treatment (Fig. 5B), reinforcing their role in metastasis. Although AS treatment reduced CTCs with McTNs (mean per patient: 20 vs. 15 cells), this reduction was not statistically significant (Table S5, *p* = 0.250).

AS treatment significantly reduced total CTC counts (*p* < 0.001, Fig. 6A and Table S3), aligning with its cytotoxic effects in cell lines. Reducing CTC burden is significant, as CTC counts correlate with metastatic progression and poor prognosis [16,17].

To assess whether AS acts through apoptotic pathways in CTCs, resulting in the eventual reduction of total tumor cells, M30/CD45 staining was performed before and after AS treatment of patients’ samples (Fig. 6B). Apoptotic (CK+/M30+/CD45–) CTCs increased significantly following AS treatment (*p* = 0.021, Table S4), suggesting that AS effectively promotes apoptosis in CTCs. Conversely, non-apoptotic (CK+/M30–/CD45–) CTCs decreased, albeit not significantly. This increase in apoptotic M30+ CTCs highlights the ability of AS to target viable CTCs. By promoting apoptosis in CTCs, AS may serve as a potential therapeutic strategy for limiting metastatic dissemination. Future experiments could also include more apoptotic markers to reinforce these results.

Phenotypic characterization through CK/PD-L1/CD45 staining (Fig. 5D) identified PD-L1-positive CTCs, in patients’ samples both before and after AS treatment, reinforcing the role of this immune checkpoint molecule in aiding CTC immune evasion [18,19,20]. AS reduced PD-L1-positive CTCs, however, not in a statistically significant manner (Table S6). AS treatment also significantly reduced specific CTC subclones (Fig. 6C, Table S6), including (CK+/CXCR4+/JUNB−; *p* = 0.01) and (CK+/VIM+/GLU+; *p* < 0.001), suggesting that AS may disrupt CXCR4-mediated chemotaxis. CXCR4 supports CTC extravasation and organ-specific metastasis through C-X-C motif chemokine ligand 12 (CXCL12) signaling [61], and its downregulation may impair metastatic colonization. These results are interesting as the expression of CXCR4 in CTCs has emerged as a poor prognostic factor for these patients [16]. The observed reduction in (CK+/VIM+/GLU+) CTCs suggests that AS interferes with EMT-driven plasticity, which is strongly linked to invasiveness and apoptosis resistance. This phenotype has been linked to poor prognosis when expressed in patients’ CTCs with breast cancer and NSCLC [21,22]. CK/VIM/GLU staining (Fig. 5F) confirmed the presence of VIM+ and GLU+ CTCs prior to AS treatment, consistent with an EMT-like phenotype known to enhance metastatic capacity [19,21,22,62,63]. VIM, a marker of mesenchymal transition, promotes cell plasticity and metastatic behavior [64]. Similarly, GLU stabilizes microtubule networks, enhancing cell flexibility and migration, crucial for CTC survival in circulation [21]. By reducing these markers, AS may impair key survival mechanisms that enable CTCs to persist in circulation and evade immune detection. These findings highlight AS’s ability to target aggressive CTC subpopulations, potentially restricting their metastatic ability. Further focus on the pathways involved in AS effect on CTCs will be important for future studies.

Finally, a limitation of this study is the use of different biological entities to explore AS’s effect on metastatic dissemination, such as cancer cell lines, the CTC-MCC-41 cell line, and patient-derived CTCs. However, interestingly, AS was effective on all these entities, reinforcing the significance of these results. AS emerges as a promising potential modulator of metastasis-associated processes by interfering with pathways linked to EMT, and apoptosis resistance. Further *in-vivo* studies using patient-derived CTCs from various cancers are warranted to confirm AS’s efficacy and establish its anti-metastatic potential. Future research should also explore its molecular mechanisms and potential in combination therapies to improve disease outcomes. In addition, based on the consistency of the results between non-adherent cells, CTC-MCC-41 cells, and CTCs, this study provides a path for the evaluation of new drugs for metastatic dissemination, starting from cell line models and continuing with valuable clinical samples.

## 5 Conclusions

This study demonstrates the anti-metastatic efficacy of AS across various cancer models, including adherent and anoikis-resistant cell lines, as well as patient-derived CTCs from SCLC patients. AS showed strong cytotoxic effects against aggressive tumor subpopulations, while having minimal impact on normal epithelial cells. It was also associated with reduced CTC viability, modulation of EMT-related markers, and induction of apoptosis. AS’s ability to target metastasis-competent CTCs is significant, as these cells drive tumor spread and therapy resistance. Further studies in a larger cohort of patients will help strengthen these results.

## Data Availability

The authors confirm that the data supporting the findings of this study are available within the article and/or its Supplementary Materials.

## Supplementary Materials

The supplementary material is available online at https://www.techscience.com/doi/10.32604/or.2026.075600/s1.

## References

[1] Dianat-Moghadam H , Azizi M , Eslami SZ , Cortés-Hernández LE , Heidarifard M , Nouri M , et al. The role of circulating tumor cells in the metastatic cascade: Biology, technical challenges, and clinical relevance. Cancers. 2020; 12( 4): 867. doi:10.3390/cancers12040867. 32260071 PMC7225923

[2] Dujon AM , Capp JP , Brown JS , Pujol P , Gatenby RA , Ujvari B , et al. Is there one key step in the metastatic cascade? Cancers. 2021; 13( 15): 3693. doi:10.3390/cancers13153693. 34359593 PMC8345184

[3] Alix-Panabières C , Pantel K . Challenges in circulating tumour cell research. Nat Rev Cancer. 2014; 14( 9): 623– 31. doi:10.1038/nrc3820. 25154812

[4] Adeshakin FO , Adeshakin AO , Afolabi LO , Yan D , Zhang G , Wan X . Mechanisms for modulating anoikis resistance in cancer and the relevance of metabolic reprogramming. Front Oncol. 2021; 11: 626577. doi:10.3389/fonc.2021.626577. 33854965 PMC8039382

[5] Atjanasuppat K , Lirdprapamongkol K , Jantaree P , Svasti J . Non-adherent culture induces paclitaxel resistance in H460 lung cancer cells via ERK-mediated up-regulation of βIVa-tubulin. Biochem Biophys Res Commun. 2015; 466( 3): 493– 8. doi:10.1016/j.bbrc.2015.09.057. 26375501

[6] Yang C , Liu Y , Gai L , Zhang Z , Zhang Y , Zhang G , et al. Artesunate regulates malignant progression of breast cancer cells via lncRNA TUG1/miR-145-5p/HOXA5 axis. PLoS One. 2025; 20( 8): e0329490. doi:10.1371/journal.pone.0329490. 40758729 PMC12321065

[7] Pirali M , Taheri M , Zarei S , Majidi M , Ghafouri H . Artesunate, as a HSP70 ATPase activity inhibitor, induces apoptosis in breast cancer cells. Int J Biol Macromol. 2020; 164: 3369– 75. doi:10.1016/j.ijbiomac.2020.08.198. 32861782

[8] Zhang Y , Wang Y , Li Y , Huang C , Xiao X , Zhong Z , et al. Dihydroartemisinin and artesunate inhibit aerobic glycolysis via suppressing c-Myc signaling in non-small cell lung cancer. Biochem Pharmacol. 2022; 198: 114941. doi:10.1016/j.bcp.2022.114941. 35149053

[9] Huang Z , Gan S , Zhuang X , Chen Y , Lu L , Wang Y , et al. Artesunate inhibits the cell growth in colorectal cancer by promoting ROS-dependent cell senescence and autophagy. Cells. 2022; 11( 16): 2472. doi:10.3390/cells11162472. 36010550 PMC9406496

[10] Fan X , Yan Y , Li Y , Song Y , Li B . Anti-tumor mechanism of artesunate. Front Pharmacol. 2024; 15: 1483049. doi:10.3389/fphar.2024.1483049. 39525639 PMC11549674

[11] Ishikawa C , Mori N . The anti-malaria agent artesunate exhibits cytotoxic effects in primary effusion lymphoma. Investig New Drugs. 2021; 39( 1): 111– 21. doi:10.1007/s10637-020-00996-1. 32885355

[12] Sundqvist A , Morikawa M , Ren J , Vasilaki E , Kawasaki N , Kobayashi M , et al. JUNB governs a feed-forward network of TGFβ signaling that aggravates breast cancer invasion. Nucleic Acids Res. 2018; 46( 3): 1180– 95. doi:10.1093/nar/gkx1190. 29186616 PMC5814809

[13] Gervasi M , Bianchi-Smiraglia A , Cummings M , Zheng Q , Wang D , Liu S , et al. JunB contributes to Id2 repression and the epithelial-mesenchymal transition in response to transforming growth factor-β. J Cell Biol. 2012; 196( 5): 589– 603. doi:10.1083/jcb.201109045. 22391036 PMC3307698

[14] Green MR , Rodig S , Juszczynski P , Ouyang J , Sinha P , O’Donnell E , et al. Constitutive AP-1 activity and EBV infection induce PD-L1 in Hodgkin lymphomas and posttransplant lymphoproliferative disorders: Implications for targeted therapy. Clin Cancer Res. 2012; 18( 6): 1611– 8. doi:10.1158/1078-0432.CCR-11-1942. 22271878 PMC3321508

[15] Wang JS , Wang MJ , Lu X , Zhang J , Liu QX , Zhou D , et al. Artesunate inhibits epithelial-mesenchymal transition in non-small-cell lung cancer (NSCLC) cells by down-regulating the expression of BTBD7. Bioengineered. 2020; 11( 1): 1197– 207. doi:10.1080/21655979.2020.1834727. 33108235 PMC8291784

[16] Roumeliotou A , Pantazaka E , Xagara A , Dimitrakopoulos FI , Koutras A , Christopoulou A , et al. Phenotypic characterization of circulating tumor cells isolated from non-small and small cell lung cancer patients. Cancers. 2022; 15( 1): 171. doi:10.3390/cancers15010171. 36612166 PMC9818148

[17] Papakonstantinou D , Roumeliotou A , Pantazaka E , Shaukat AN , Christopoulou A , Koutras A , et al. Integrative analysis of circulating tumor cells (CTCs) and exosomes from small-cell lung cancer (SCLC) patients: A comprehensive approach. Mol Oncol. 2025; 19( 7): 2038– 55. doi:10.1002/1878-0261.13765. 39575761 PMC12234381

[18] Xagara A , Roumeliotou A , Kokkalis A , Tsapakidis K , Papakonstantinou D , Papadopoulos V , et al. ES-SCLC patients with PD-L1^+^ CTCs and high percentages of CD8^+^PD-1^+^T cells in circulation benefit from front-line immunotherapy treatment. Biomedicines. 2024; 12( 1): 146. doi:10.3390/biomedicines12010146. 38255251 PMC10813758

[19] Vardas V , Tolios A , Christopoulou A , Georgoulias V , Xagara A , Koinis F , et al. Immune checkpoint and EMT-related molecules in circulating tumor cells (CTCs) from triple negative breast cancer patients and their clinical impact. Cancers. 2023; 15( 7): 1974. doi:10.3390/cancers15071974. 37046635 PMC10093450

[20] Eslami SZ , Cortés-Hernández LE , Sinoquet L , Gauthier L , Vautrot V , Cayrefourcq L , et al. Circulating tumour cells and PD-L1-positive small extracellular vesicles: The liquid biopsy combination for prognostic information in patients with metastatic non-small cell lung cancer. Br J Cancer. 2024; 130( 1): 63– 72. doi:10.1038/s41416-023-02491-9. 37973956 PMC10781977

[21] Katsarou SD , Messaritakis I , Voumvouraki A , Kakavogiannis S , Kotsakis A , Alkahtani S , et al. Detyrosinated A-tubulin, vimentin and PD-L1 in circulating tumor cells (CTCs) isolated from non-small cell lung cancer (NSCLC) patients. J Pers Med. 2022; 12( 2): 154. doi:10.3390/jpm12020154. 35207643 PMC8875112

[22] Kallergi G , Aggouraki D , Zacharopoulou N , Stournaras C , Georgoulias V , Martin SS . Evaluation of α-tubulin, detyrosinated α-tubulin, and vimentin in CTCs: Identification of the interaction between CTCs and blood cells through cytoskeletal elements. Breast Cancer Res. 2018; 20( 1): 67. doi:10.1186/s13058-018-0993-z. 29976237 PMC6034292

[23] Cao D , Chen D , Xia JN , Wang WY , Zhu GY , Chen LW , et al. Artesunate promoted anti-tumor immunity and overcame EGFR-TKI resistance in non-small-cell lung cancer by enhancing oncogenic TAZ degradation. Biomed Pharmacother. 2022; 155: 113705. doi:10.1016/j.biopha.2022.113705. 36271541

[24] Cayrefourcq L , Mazard T , Joosse S , Solassol J , Ramos J , Assenat E , et al. Establishment and characterization of a cell line from human circulating colon cancer cells. Cancer Res. 2015; 75( 5): 892– 901. doi:10.1158/0008-5472.CAN-14-2613. 25592149

[25] Ju JA , Lee CJ , Thompson KN , Ory EC , Lee RM , Mathias TJ , et al. Partial thermal imidization of polyelectrolyte multilayer cell tethering surfaces (TetherChip) enables efficient cell capture and microtentacle fixation for circulating tumor cell analysis. Lab Chip. 2020; 20( 16): 2872– 88. doi:10.1039/D0LC00207K. 32744284 PMC7595763

[26] Matrone MA , Whipple RA , Balzer EM , Martin SS . Microtentacles tip the balance of cytoskeletal forces in circulating tumor cells. Cancer Res. 2010; 70( 20): 7737– 41. doi:10.1158/0008-5472.CAN-10-1569. 20924109 PMC4232206

[27] Presser A , Feichtinger A , Buzzi S . A simplified and scalable synthesis of artesunate. Monatsh Chem. 2017; 148( 1): 63– 8. doi:10.1007/s00706-016-1865-9. 28127092 PMC5225229

[28] Zhao F , Klimecki WT . Culture conditions profoundly impact phenotype in BEAS-2B, a human pulmonary epithelial model. J Appl Toxicol. 2015; 35( 8): 945– 51. doi:10.1002/jat.3094. 25524072 PMC4474793

[29] Soler A , Cayrefourcq L , Mazard T , Babayan A , Lamy PJ , Assou S , et al. Autologous cell lines from circulating colon cancer cells captured from sequential liquid biopsies as model to study therapy-driven tumor changes. Sci Rep. 2018; 8( 1): 15931. doi:10.1038/s41598-018-34365-z. 30374140 PMC6206091

[30] Pateras IS , Williams C , Gianniou DD , Margetis AT , Avgeris M , Rousakis P , et al. Short term starvation potentiates the efficacy of chemotherapy in triple negative breast cancer via metabolic reprogramming. J Transl Med. 2023; 21( 1): 169. doi:10.1186/s12967-023-03935-9. 36869333 PMC9983166

[31] Balcik-Ercin P , Cayrefourcq L , Soundararajan R , Mani SA , Alix-Panabières C . Epithelial-to-mesenchymal plasticity in circulating tumor cell lines sequentially derived from a patient with colorectal cancer. Cancers. 2021; 13( 21): 5408. doi:10.3390/cancers13215408. 34771571 PMC8582537

[32] Papadaki MA , Kallergi G , Zafeiriou Z , Manouras L , Theodoropoulos PA , Mavroudis D , et al. Co-expression of putative stemness and epithelial-to-mesenchymal transition markers on single circulating tumour cells from patients with early and metastatic breast cancer. BMC Cancer. 2014; 14: 651. doi:10.1186/1471-2407-14-651. 25182808 PMC4161777

[33] Kallergi G , Politaki E , Alkahtani S , Stournaras C , Georgoulias V . Evaluation of isolation methods for circulating tumor cells (CTCs). Cell Physiol Biochem. 2016; 40( 3–4): 411– 9. doi:10.1159/000452556. 27889762

[34] Choe C , Kim H , Min S , Park S , Seo J , Roh S . *SOX2*, a stemness gene, induces progression of NSCLC A549 cells toward anchorage-independent growth and chemoresistance to vinblastine. Onco Targets Ther. 2018; 11: 6197– 207. doi:10.2147/OTT.S175810. 30288055 PMC6163012

[35] Khaw-On P , Pompimon W , Banjerdpongchai R . Goniothalamin induces necroptosis and anoikis in human invasive breast cancer MDA-MB-231 cells. Int J Mol Sci. 2019; 20( 16): 3953. doi:10.3390/ijms20163953. 31416203 PMC6720804

[36] Magoulas GE , Tsigkou T , Skondra L , Lamprou M , Tsoukala P , Kokkinogouli V , et al. Synthesis of novel artemisinin dimers with polyamine linkers and evaluation of their potential as anticancer agents. Bioorg Med Chem. 2017; 25( 14): 3756– 67. doi:10.1016/j.bmc.2017.05.018. 28549888

[37] Vardas V , Ju JA , Christopoulou A , Xagara A , Georgoulias V , Kotsakis A , et al. Functional analysis of viable circulating tumor cells from triple-negative breast cancer patients using TetherChip technology. Cells. 2023; 12( 15): 1940. doi:10.3390/cells12151940. 37566019 PMC10416943

[38] Stemberger MB , Ju JA , Thompson KN , Mathias TJ , Jerrett AE , Chang KT , et al. Hydrogen peroxide induces α-tubulin detyrosination and acetylation and impacts breast cancer metastatic phenotypes. Cells. 2023; 12( 9): 1266. doi:10.3390/cells12091266. 37174666 PMC10177274

[39] Whipple RA , Matrone MA , Cho EH , Balzer EM , Vitolo MI , Yoon JR , et al. Epithelial-to-mesenchymal transition promotes tubulin detyrosination and microtentacles that enhance endothelial engagement. Cancer Res. 2010; 70( 20): 8127– 37. doi:10.1158/0008-5472.CAN-09-4613. 20924103 PMC3123454

[40] Kim JB , Yu JH , Ko E , Lee KW , Song AK , Park SY , et al. The alkaloid Berberine inhibits the growth of Anoikis-resistant MCF-7 and MDA-MB-231 breast cancer cell lines by inducing cell cycle arrest. Phytomedicine. 2010; 17( 6): 436– 40. doi:10.1016/j.phymed.2009.08.012. 19800775

[41] Breslin S , O’Driscoll L . The relevance of using 3D cell cultures, in addition to 2D monolayer cultures, when evaluating breast cancer drug sensitivity and resistance. Oncotarget. 2016; 7( 29): 45745– 56. doi:10.18632/oncotarget.9935. 27304190 PMC5216757

[42] Paul R , Dorsey JF , Fan Y . Cell plasticity, senescence, and quiescence in cancer stem cells: Biological and therapeutic implications. Pharmacol Ther. 2022; 231: 107985. doi:10.1016/j.pharmthera.2021.107985. 34480963 PMC8844041

[43] Qiu F , Liu J , Mo X , Liu H , Chen Y , Dai Z . Immunoregulation by artemisinin and its derivatives: A new role for old antimalarial drugs. Front Immunol. 2021; 12: 751772. doi:10.3389/fimmu.2021.751772. 34567013 PMC8458561

[44] Chen L , Wang J , Ren Y , Ma Y , Liu J , Jiang H , et al. Artesunate improves glucose and lipid metabolism in db/db mice by regulating the metabolic profile and the MAPK/PI3K/Akt signalling pathway. Phytomedicine. 2024; 126: 155382. doi:10.1016/j.phymed.2024.155382. 38382280

[45] Zhao C , Kong X , Han S , Li X , Wu T , Zhou J , et al. Analysis of differential metabolites in lung cancer patients based on metabolomics and bioinformatics. Future Oncol. 2020; 16( 18): 1269– 87. doi:10.2217/fon-2019-0818. 32356461

[46] Hill KS , McDowell A , McCorkle JR , Schuler E , Ellingson SR , Plattner R , et al. KEAP1 is required for artesunate anticancer activity in non-small-cell lung cancer. Cancers. 2021; 13( 8): 1885. doi:10.3390/cancers13081885. 33920029 PMC8070990

[47] Rassias DJ , Weathers PJ . Dried leaf *Artemisia annua* efficacy against non-small cell lung cancer. Phytomedicine. 2019; 52: 247– 53. doi:10.1016/j.phymed.2018.09.167. 30599905

[48] Hu P , Ni C , Teng P . Effects of artesunate on the malignant biological behaviors of non-small cell lung cancer in human and its mechanism. Bioengineered. 2022; 13( 3): 6590– 9. doi:10.1080/21655979.2022.2042141. 35361045 PMC9278965

[49] Fröhlich T , Kiss A , Wölfling J , Mernyák E , Kulmány ÁE , Minorics R , et al. Synthesis of artemisinin-estrogen hybrids highly active against HCMV, P. falciparum, and cervical and breast cancer. ACS Med Chem Lett. 2018; 9( 11): 1128– 33. doi:10.1021/acsmedchemlett.8b00381. 30429957 PMC6231177

[50] Wen L , Liu L , Wen L , Yu T , Wei F . Artesunate promotes G2/M cell cycle arrest in MCF7 breast cancer cells through ATM activation. Breast Cancer. 2018; 25( 6): 681– 6. doi:10.1007/s12282-018-0873-5. 29797234

[51] Duarte D , Nunes M , Ricardo S , Vale N . Combination of antimalarial and CNS drugs with antineoplastic agents in MCF-7 breast and HT-29 colon cancer cells: Biosafety evaluation and mechanism of action. Biomolecules. 2022; 12( 10): 1490. doi:10.3390/biom12101490. 36291699 PMC9599492

[52] Sethy C , Kundu CN . 5-Fluorouracil (5-FU) resistance and the new strategy to enhance the sensitivity against cancer: Implication of DNA repair inhibition. Biomed Pharmacother. 2021; 137: 111285. doi:10.1016/j.biopha.2021.111285. 33485118

[53] Alzahrani SM , Al Doghaither HA , Al-Ghafari AB , Pushparaj PN . 5-Fluorouracil and capecitabine therapies for the treatment of colorectal cancer (review). Oncol Rep. 2023; 50( 4): 175. doi:10.3892/or.2023.8612. 37594133

[54] Azimi S , Esmaeil Lashgarian H , Ghorbanzadeh V , Moradipour A , Pirzeh L , Dariushnejad H . 5-FU and the dietary flavonoid carvacrol: A synergistic combination that induces apoptosis in MCF-7 breast cancer cells. Med Oncol. 2022; 39( 12): 253. doi:10.1007/s12032-022-01863-0. 36224408

[55] Mathew AA , Zakkariya ZT , Ashokan A , Manohar M , Keechilat P , Nair SV , et al. 5-FU mediated depletion of myeloid suppressor cells enhances T-cell infiltration and anti-tumor response in immunotherapy-resistant lung tumor. Int Immunopharmacol. 2023; 120: 110129. doi:10.1016/j.intimp.2023.110129. 37201402

[56] Gillet JP , Varma S , Gottesman MM . The clinical relevance of cancer cell lines. J Natl Cancer Inst. 2013; 105( 7): 452– 8. doi:10.1093/jnci/djt007. 23434901 PMC3691946

[57] Cohen EN , Jayachandran G , Moore RG , Cristofanilli M , Lang JE , Khoury JD , et al. A multi-center clinical study to harvest and characterize circulating tumor cells from patients with metastatic breast cancer using the parsortix^®^ PC1 system. Cancers. 2022; 14( 21): 5238. doi:10.3390/cancers14215238. 36358657 PMC9656921

[58] Maertens Y , Humberg V , Erlmeier F , Steffens S , Steinestel J , Bögemann M , et al. Comparison of isolation platforms for detection of circulating renal cell carcinoma cells. Oncotarget. 2017; 8( 50): 87710– 7. doi:10.18632/oncotarget.21197. 29152114 PMC5675666

[59] Guo Z , Xia W . Isolation of circulating tumor cells: Recent progress and future perspectives. Med X. 2024; 2( 1): 28. doi:10.1007/s44258-024-00044-0.

[60] Yang X , Zheng Y , Liu L , Huang J , Wang F , Zhang J . Progress on the study of the anticancer effects of artesunate. Oncol Lett. 2021; 22( 5): 750. doi:10.3892/ol.2021.13011. 34539854 PMC8436334

[61] Martinez-Ordoñez A , Seoane S , Cabezas P , Eiro N , Sendon-Lago J , Macia M , et al. Breast cancer metastasis to liver and lung is facilitated by Pit-1-CXCL12-CXCR4 axis. Oncogene. 2018; 37( 11): 1430– 44. doi:10.1038/s41388-017-0036-8. 29321662

[62] Lindsay CR , Le Moulec S , Billiot F , Loriot Y , Ngo-Camus M , Vielh P , et al. Vimentin and Ki67 expression in circulating tumour cells derived from castrate-resistant prostate cancer. BMC Cancer. 2016; 16: 168. doi:10.1186/s12885-016-2192-6. 26923772 PMC4770547

[63] Yu J , Yang M , Peng T , Liu Y , Cao Y . Evaluation of cell surface vimentin positive circulating tumor cells as a prognostic biomarker for stage III/IV colorectal cancer. Sci Rep. 2023; 13( 1): 18791. doi:10.1038/s41598-023-45951-1. 37914786 PMC10620146

[64] Satelli A , Li S . Vimentin in cancer and its potential as a molecular target for cancer therapy. Cell Mol Life Sci. 2011; 68( 18): 3033– 46. doi:10.1007/s00018-011-0735-1. 21637948 PMC3162105

