# Supplementary material for: Repurposing Artesunate to Combat Progression and Metastasis via Targeting Circulating Tumor Cells
Source: Oncol Res. 2026 May 21;34(6):13. doi: 10.32604/or.2026.075600 (PMC13223190; doi:10.32604/or.2026.075600)
Supplement: Supplementary file 1 [file OncolRes-34-75600-s001.zip › TSP_OR_75600-s001.docx]

**Supplementary Table S1:** Decrease (%) of the viability of adherent and non-adherent cells after treatment for 48 h with the indicated drugs and concentrations.

| **Tissue** | **Cancer Cell Line** | **Treatment** | **Adherent, -PolyHEMA-Treated Cells** | **Non-Adherent, +PolyHEMA-Treated Cells** | ***p*^#^** | **Difference** |
| --- | --- | --- | --- | --- | --- | --- |
| Lung | H1299 | AS (10 μΜ) | 63 ± 0.5 (4) | 28 ± 6 (4) | * | 35 |
|  |  | AS (100 μΜ) | 100 ± 0 (4) | 99 ± 1 (4) | ns | 1 |
|  |  | 5-FU (10 μΜ) | 8 ± 2 (3) | 15 ± 1 (3) | ns | −7 |
|  | A549 | AS (10 μΜ) | 43 ± 13 (4) | 18 ± 8 (4) | * | 25 |
|  |  | AS (100 μΜ) | 94 ± 1 (4) | 68 ± 8 (4) | * | 26 |
|  |  | 5-FU (10 μΜ) | 38 ± 2 (4) | 30 ± 4 (4) | ns | 8 |
|  | DMS 454 | AS (10 μΜ) | 56 ± 5 (3) | 34 ± 4 (3) | * | 22 |
|  |  | AS (100 μΜ) | 80 ± 2 (3) | 60 ± 6 (3) | * | 20 |
|  |  | 5-FU (10 μΜ) | 36 ± 4 (3) | 29 ± 5 (3) | ns | 7 |
|  | BEAS-2B | AS (10 μΜ) | 22 ± 7 (4) | 53 ± 23 (2) | - | −31 |
|  |  | AS (100 μΜ) | 66 ± 5 (4) | 69 ± 4 (2) | - | −3 |
|  |  | 5-FU (10 μΜ) | 12 ± 2 (4) | 15 ± 17 (2) | - | −3 |
| Breast | MDA-MB-231 | AS (10 μΜ) | 49 ± 3 (3) | 15 ± 8 (3) | * | 34 |
|  |  | AS (100 μΜ) | 91 ± 2 (3) | 69 ± 4 (3) | * | 22 |
|  |  | 5-FU (10 μΜ) | 5 ± 5 (3) | 15 ± 2 (3) | ns | −10 |
|  | MDA-MB-436 | AS (10 μΜ) | 48 ± 4 (4) | 15 ± 1 (3) | ns | 33 |
|  |  | AS (100 μΜ) | 89 ± 2 (4) | 55 ± 6 (3) | ns | 34 |
|  |  | 5-FU (10 μΜ) | 42 ± 0 (2) | 32 ± 23 (2) | - | 10 |
|  | MCF-7 | AS (10 μΜ) | 40 ± 8 (4) | 24 ± 5 (4) | ns | 16 |
|  |  | AS (100 μΜ) | 95 ± 1 (4) | 56 ± 5 (4) | ** | 39 |
|  |  | 5-FU (10 μΜ) | 27 ± 4 (4) | 22 ± 7 (4) | ns | 5 |
| Colon | HT-29 | AS (10 μΜ) | 57 ± 3 (4) | 36 ± 7 (4) | ns | 21 |
|  |  | AS (100 μΜ) | 88 ± 1 (4) | 65 ± 9 (4) | ns | 23 |
|  |  | 5-FU (10 μΜ) | 26 ± 10 (2) | 13 ± 5 (2) | c | 13 |
|  | SW-620 | AS (10 μΜ) | 42 ± 3 (4) | 43 ± 6 (4) | ns | −1 |
|  |  | AS (100 μΜ) | 60 ± 4 (4) | 65 ± 5 (4) | ns | −5 |
|  |  | 5-FU (10 μΜ) | 8 ± 16 (2) | 7 ± 13 (2) | - | 1 |
|  | CTC-MCC-41 | AS (10 μΜ) | 68 ± 3 (4) | 48 ± 3 (7) | * | 20 |
|  |  | AS (100 μΜ) | - | 92 ± 3 (7) | - | - |
|  |  | 5-FU (10 μΜ) | - | 65 ± 3 (5) | - | - |

^#^*p*-values represent statistical significance between adherent and non-adherent cells for each treatment. For *n* = 2, results are presented as mean ± range, and no statistical analysis was performed. For *n* ≥ 3, results are presented as mean ± Standard Error of the Mean (SEM). **p* < 0.05; ***p* < 0.01. Abb: AS, Artesunate; 5-FU, 5-fluorouracil.

**Supplementary Table S2:** Number of CTCs in Cytospins and Tetherchips.

| **Patients** | **Cytospins (n)** | **TetherChips (n)** |
| --- | --- | --- |
| #1 | 0 | 12 |
| #2 | 1 | 13 |
| #3 | 0 | 1 |
| #4 | 2 | 16 |
| #5 | 2 | 4 |

Abb: CTCs, Circulating Tumor Cells. n represents the number of CTCs detected per patient sample.

**Supplementary Table S3:** Number of CTCs in untreated (control, CTL) and AS-treated cells per patient*.*

| **Patients** | **Control (n)** | **AS (n)** |
| --- | --- | --- |
| #1 | 39 | 38 |
| #2 | 38 | 23 |
| #3 | 9 | 8 |
| #4 | 102 | 51 |
| #5 | 31 | 21 |

n represents the number of CTCs detected per patient sample.

**Supplementary Table S4:** Number of apoptotic (M30+) and non-apoptotic (M30–) CTCs in untreated (control, CTL) and AS-treated (10 μΜ, 24 h) cells per patient*.*

| **Patients** | **Effect** | **CK+Μ30+CD45– (n)** | **CK+Μ30–CD45– (n)** |
| --- | --- | --- | --- |
| #1 | Control  AS | 2  4 | 1  0 |
| #2 | Control  AS | 1  2 | 0  0 |
| #3 | Control  AS | 2  2 | 3  1 |
| #4 | Control  AS | 0  1 | 4  1 |
| #5 | Control  AS | 4  7 | 9  2 |

n represents the number of CTCs detected per patient sample.

**Supplementary Table S5:** Number of CTCs with or without microtentacles in untreated (control, CTL) and AS-treated cells per patient*.*

| **Patients** | **Effect** | **Tentacles (n)** | **No Tentacles (n)** |
| --- | --- | --- | --- |
| #1 | Control | 1 | 8 |
|  | AS | 0 | 8 |
| #2 | Control | 0 | 3 |
|  | AS | 0 | 3 |
| #3 | Control | 1 | 2 |
|  | AS | 1 | 2 |
| #4 | Control | 12 | 28 |
|  | AS | 6 | 10 |
| #5 | Control | 88 | 247 |
|  | AS | 70 | 195 |

n represents the number of CTCs detected per patient sample.

**Supplementary Table S6:** Number of CTCs corresponding to each phenotype in untreated (control, CTL) and AS-treated cells per patient*.*

| **Patient** | **Effect** | **CK+PD-L1+CD45– (n)** | **CK+PD-L1–CD45– (n)** | **CK+CXCR4+JUNB+ (n)** | **CK+CXCR4+JUNB– (n)** | **CK+CXCR4–JUNB+ (n)** | **CK+CXCR4–JUNB– (n)** | **CK+VIM+/GLU+ (n)** | **CK+VIM+GLU– (n)** | **CK+VIM–GLU+ (n)** | **CK+VIM–GLU– (n)** |
| --- | --- | --- | --- | --- | --- | --- | --- | --- | --- | --- | --- |
| #1 | Control | 6 | 0 | 7 | 0 | 4 | 1 | 4 | 13 | 0 | 3 |
|  | AS | 6 | 0 | 1 | 0 | 9 | 0 | 1 | 12 | 0 | 7 |
| #2 | Control | 5 | 0 | 9 | 4 | 0 | 0 | 12 | 1 | 1 | 0 |
|  | AS | 1 | 0 | 2 | 2 | 4 | 0 | 6 | 4 | 0 | 0 |
| #3 | Control | 0 | 2 | 0 | 1 | 0 | 0 | 3 | 2 | 0 | 0 |
|  | AS | 0 | 1 | 0 | 1 | 0 | 0 | 2 | 2 | 0 | 0 |
| #4 | Control | 23 | 16 | 5 | 5 | 1 | 5 | 30 | 2 | 2 | 0 |
|  | AS | 6 | 5 | 2 | 2 | 1 | 5 | 15 | 0 | 5 | 1 |
| #5 | Control | 0 | 1 | 3 | 0 | 0 | 1 | 3 | 18 | 0 | 0 |
|  | AS | 0 | 1 | 0 | 0 | 2 | 1 | 2 | 12 | 0 | 0 |

n, represents the number of CTCs detected per patient sample.


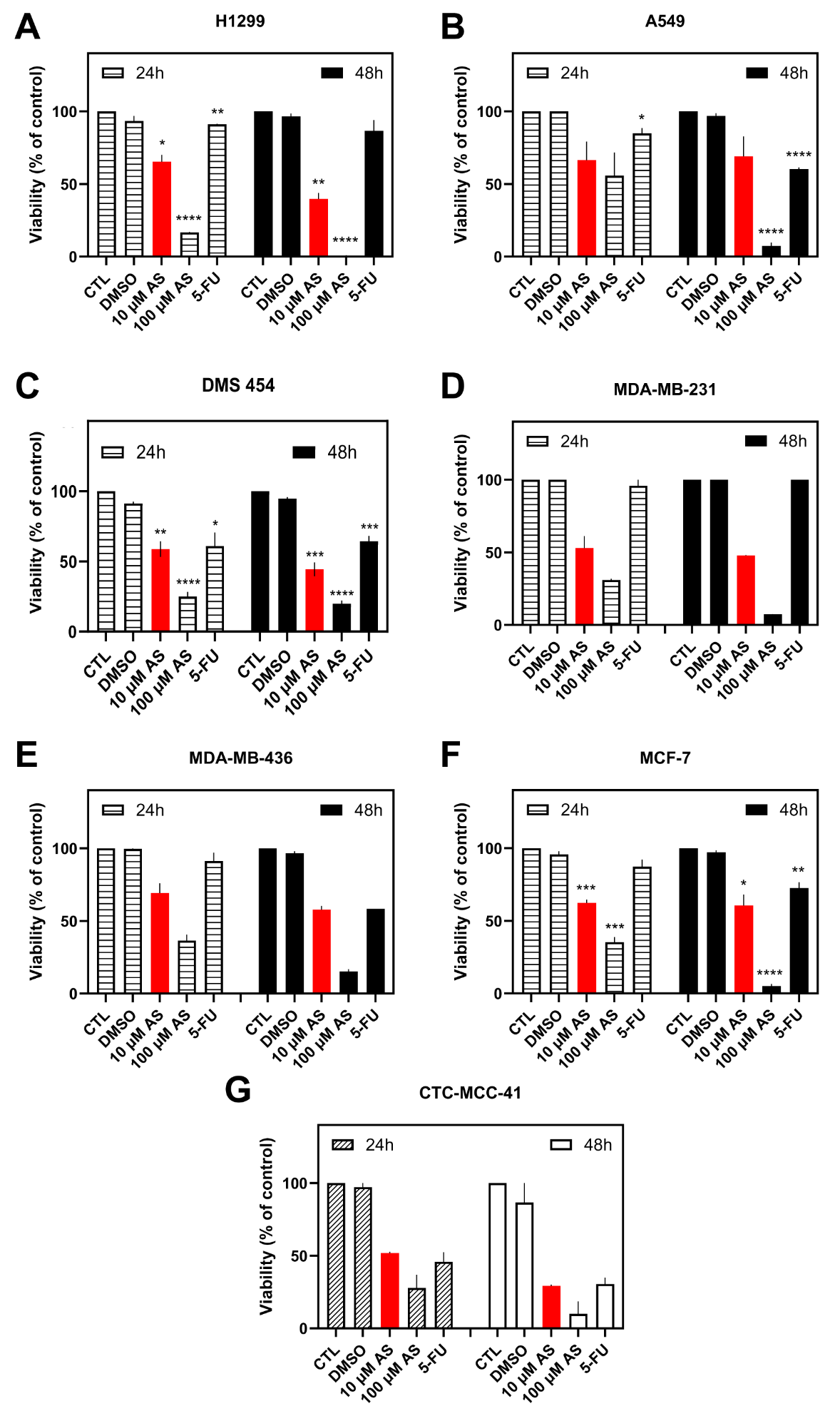


**Supplementary Figure S1:** Effect of artesunate (AS) and 5-fluorouracil (5-FU) on cells’ viability at different time points. Adherent (**A**–**F**) and non-adherent (**G**) cells treated with AS (10 and 100 μΜ), 5-FU (10 μM) and 0.1% DMSO (carrier corresponding to 100 μM AS) for 24 and 48 h. Viability (%) is normalized against untreated cells (control, CTL). Results are expressed as mean ± SEM (or mean ± range for *n* = 2) from independent experiments performed in duplicate, where **A** (*n* = 3), **B** (*n* = 4), **C** (*n* = 3), **D** (*n* = 2), **E** (*n* = 2), **F** (*n* = 4), and **G** (*n* = 2). **p* < 0.05, ***p* < 0.01, ****p* < 0.001, *****p* < 0.0001*.*


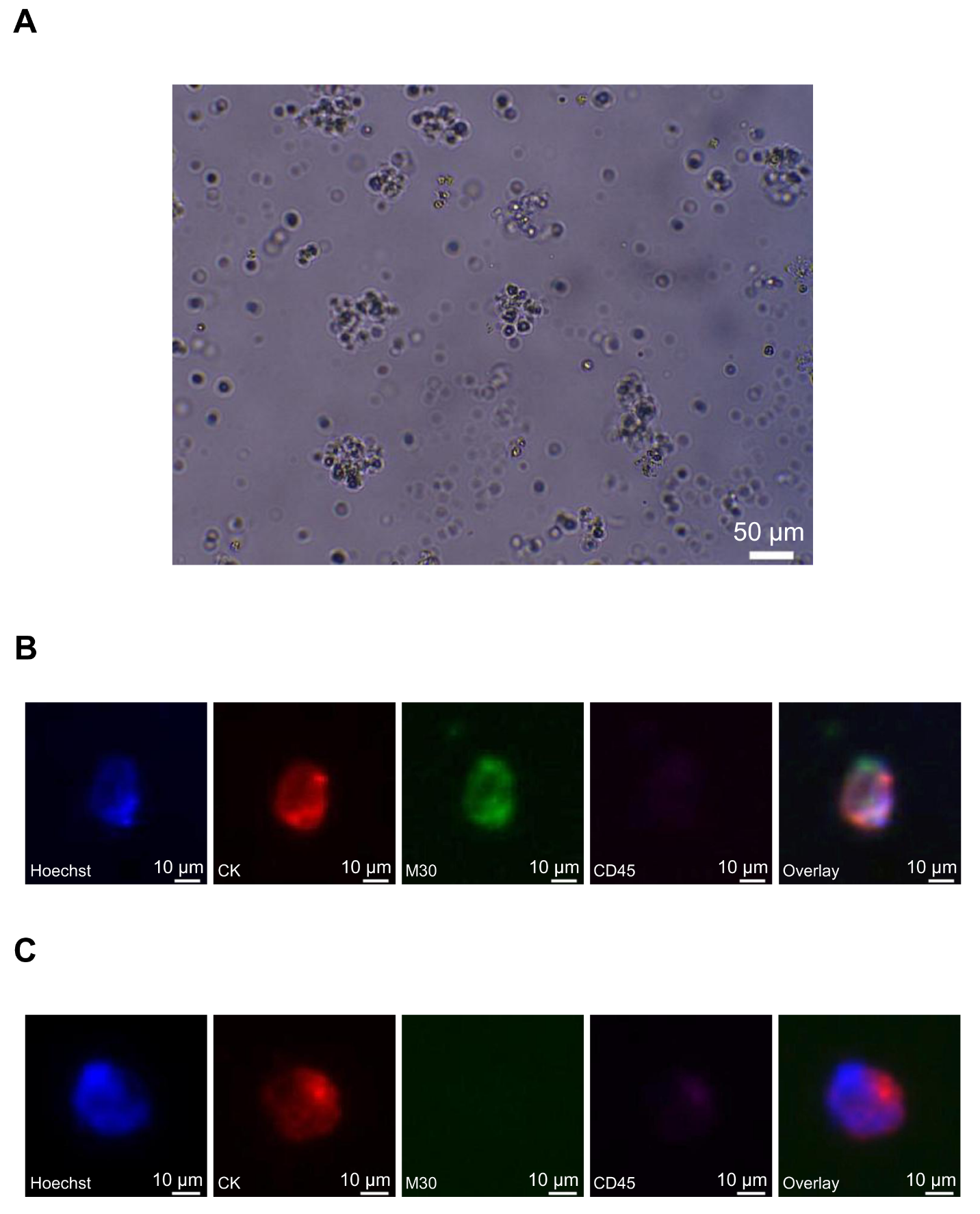


**Supplementary Figure S2:** Representative images of Small-cell lung cancer (SCLC) patient-derived circulating tumor cells (CTCs). (**A**) SCLC patient-derived CTCs after 5 days of culturing. Scale bar represents 50 μm. (**B**) SCLC patient-derived CTC stained for [Cytokeratin (CK)/M30/CD45]. Representative image of an apoptotic (CK+/M30+/CD45–) CTC. Hoechst staining of cell nuclei is in blue, CK in red, M30 in green, and CD45 in purple. Scale bars represent 10 μm. (**C**) SCLC patient-derived CTC stained for (CK)/M30/CD45). Representative image of a non-apoptotic (CK+/M30–/CD45–) CTC. Hoechst staining of cell nuclei is in blue, CK in red, M30 in green, and CD45 in purple. Scale bars represent 10 μm*.*


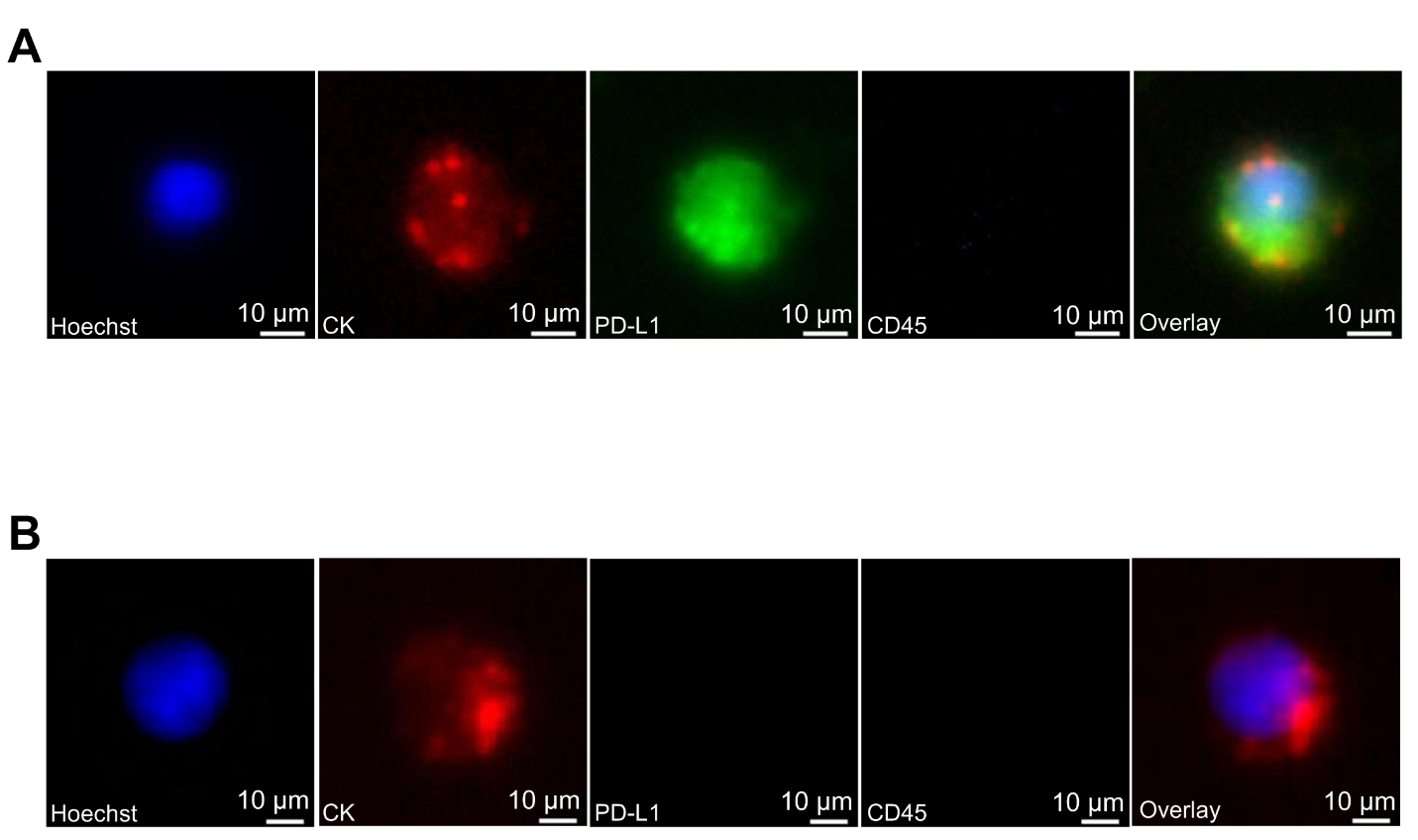


**Supplementary Figure S3:** SCLC patient-derived CTCs stained for [CK/Programmed death ligand-1 (PD-L1)/CD45]. Representative images of (**A**) a SCLC CTC expressing the CK+/PD-L1+/CD45– phenotype. (**B**) SCLC CTC expressing the CK+/PD-L1–/CD45– phenotype. Scale bars represent 10 μm*.*


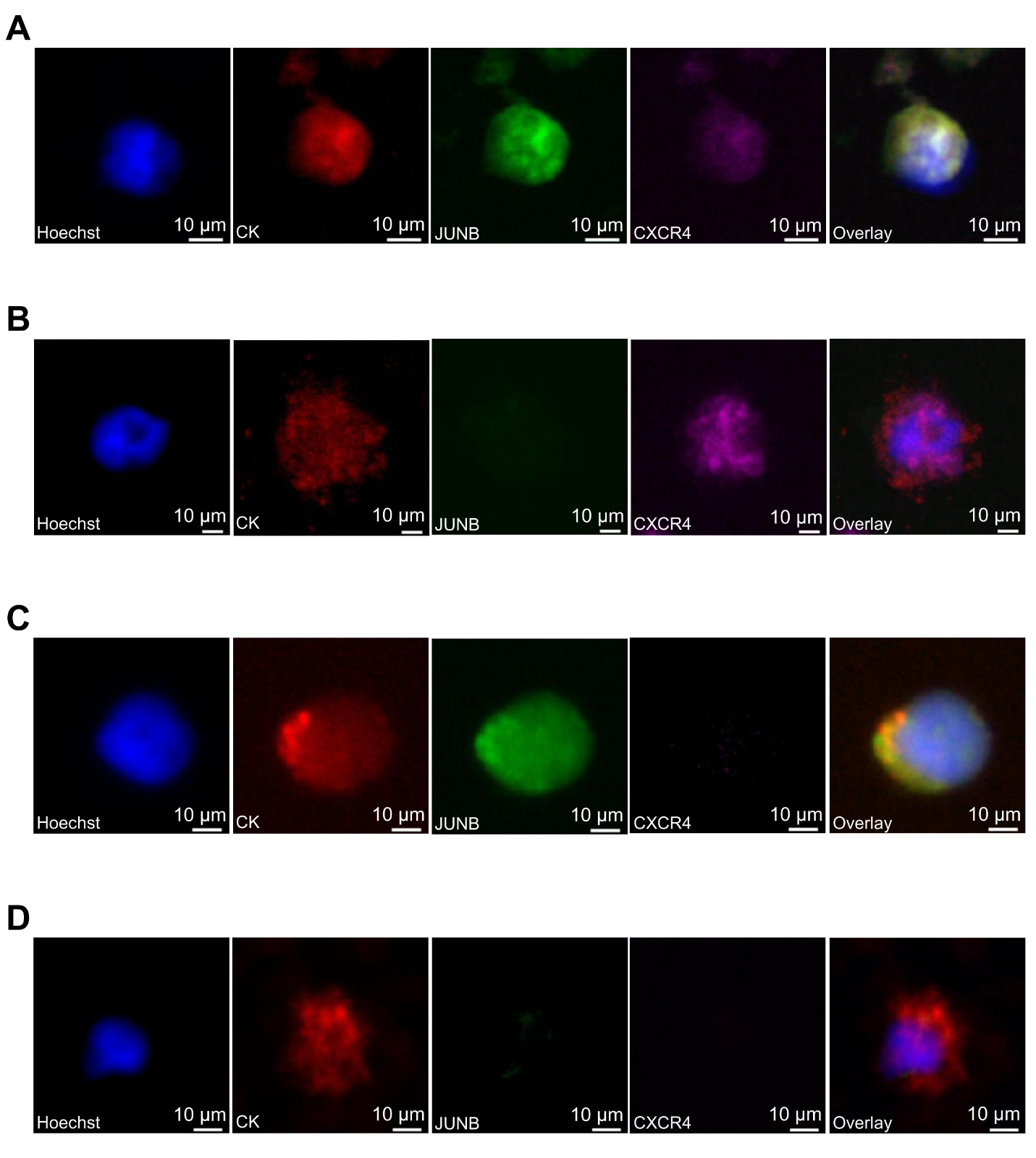


**Supplementary Figure S4:** SCLC patient-derived CTCs stained for [CK/C-X-C chemokine receptor type 4 (CXCR4)/JUNB]. Representative images of (**A**) a SCLC CTC expressing the CK+/CXCR4+/JUNB+ phenotype. (**B**) SCLC CTC expressing the CK+/CXCR4+/JUNB– phenotype. (**C**) SCLC CTC expressing the CK+/CXCR4–/JUNB+ phenotype. (**D**) SCLC CTC expressing the CK+/CXCR4–/JUNB– phenotype. Scale bars represent 10 μm*.*


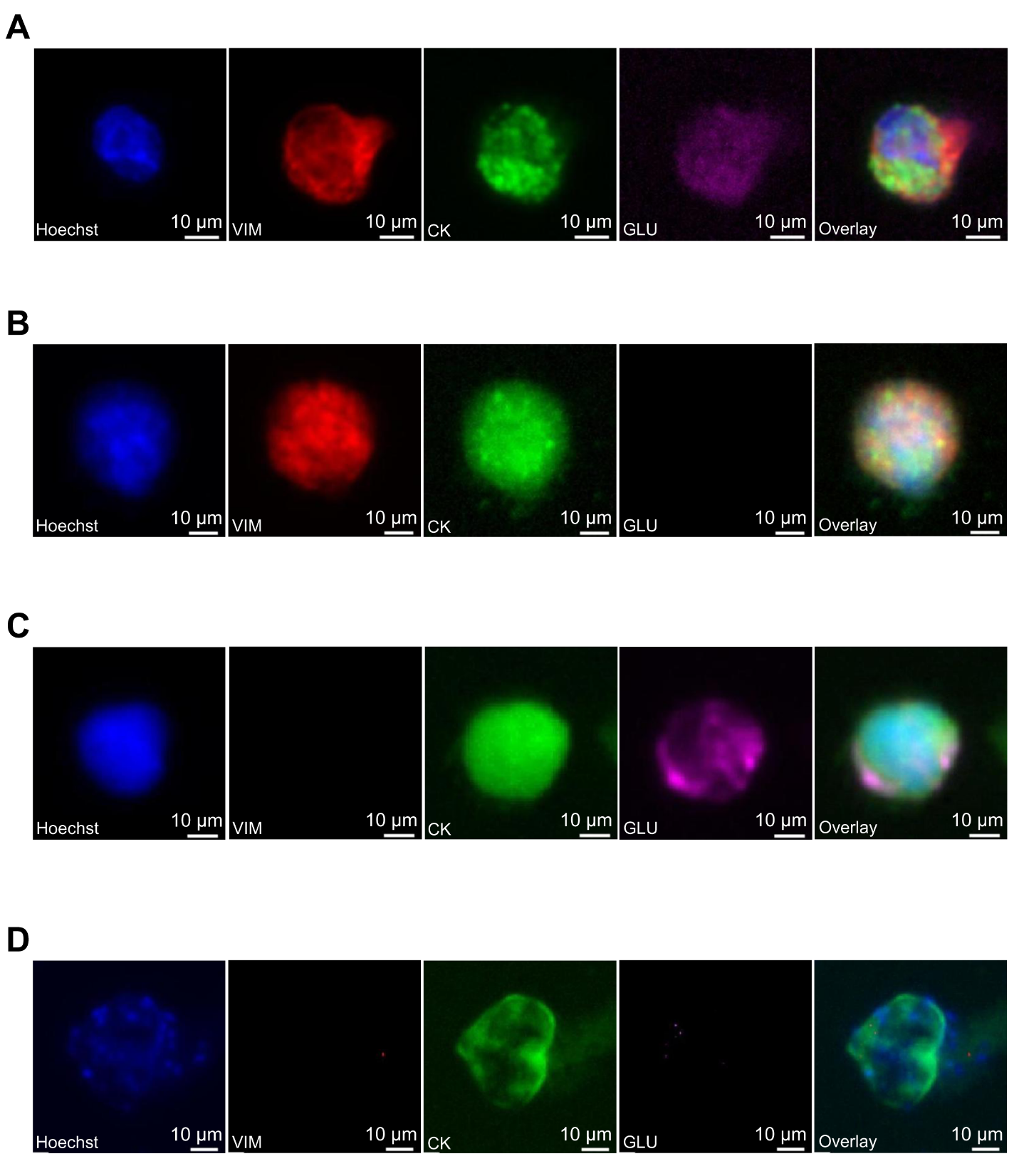


**Supplementary Figure S5:** SCLC patient-derived CTCs stained for [CK/Vimentin (VIM)/Detyrosinated a-tubulin (GLU)]. Representative images of (**A**) a SCLC CTC expressing the CK+/VIM+/GLU+ phenotype. (**B**) SCLC CTC expressing the CK+/VIM+/GLU– phenotype. (**C**) SCLC CTC expressing the CK+/VIM–/GLU+ phenotype. (**D**) SCLC CTC expressing the CK+/VIM–/GLU– phenotype. Scale bars represent 10 μm*.*
